# A clinical prediction model integrating cardiovascular-kidney-metabolic biomarkers for the composite outcome of quality-of-life deterioration and rehospitalization in elderly HFpEF patients

**DOI:** 10.3389/fendo.2026.1861971

**Published:** 2026-08-03

**Authors:** Chen He, Haimei Zhao, Heng Liu, Xiaodong Zhao, Mingting Zhang, Jinfeng Li

**Affiliations:** 1 Department of Ultrasound Medicine, Zhangjiakou First Hospital, Zhangjiakou, China; 2 Department of Cardiology, Zhangjiakou First Hospital, Zhangjiakou, China; 3 Administrative Office, Zhangjiakou First Hospital, Zhangjiakou, China

**Keywords:** cardiovascular-kidney-metabolic syndrome, diastolic dysfunction, glycated haemoglobin A1c, heart failure with preserved ejection fraction, nomogram, quality of life, risk prediction, temporal validation

## Abstract

**Background:**

Cardiovascular-kidney-metabolic (CKM) syndrome stage 4a encompasses elderly heart failure with preserved ejection fraction (HFpEF) patients with metabolic risk factors or chronic kidney disease (CKD). No existing HFpEF prediction tool has incorporated glycated haemoglobin A1c (HbA1c) alongside echocardiographic diastolic function parameters within a CKM-anchored architecture targeting quality-of-life (QoL) deterioration.

**Objective:**

To develop, internally validate, and temporally validate a least absolute shrinkage and selection operator (LASSO)-Cox nomogram for predicting the long-term composite endpoint of quality-of-life (QoL) deterioration and unplanned heart failure rehospitalisation in elderly CKM stage 4a HFpEF patients.

**Methods:**

We retrospectively enrolled 302 consecutive patients aged ≥60 years with HFpEF meeting CKM stage 4a criteria admitted between January 2020 and December 2023, and a separate temporal validation cohort (n = 102) admitted between January 2024 and June 2025. Eligible patients had confirmed HFpEF per 2021 European Society of Cardiology guidelines (symptomatic heart failure [New York Heart Association {NYHA} class II–IV], left ventricular ejection fraction [LVEF] ≥50%, elevated N-terminal pro-B-type natriuretic peptide [NT-proBNP], and echocardiographic diastolic dysfunction) and fulfilled CKM stage 4a criteria (metabolic risk factors or CKD without dialysis dependency). The primary outcome was a composite of QoL deterioration (Minnesota Living with Heart Failure Questionnaire [MLHFQ] increase ≥5 points) and unplanned heart failure rehospitalisation. Feature selection used LASSO-Cox regression integrated with multiple imputation.

**Results:**

The primary endpoint occurred in 116 patients (38.4%) over a median follow-up of 22.4 months. LASSO selected eight predictors: age, NYHA class, ln-NT-proBNP, estimated glomerular filtration rate (eGFR), uric acid, E/e′ ratio, left atrial volume index (LAVI), and HbA1c; LVEF was compressed to zero. The nomogram achieved C-indices of 0.812 (95% CI 0.770–0.854) in training, 0.790 (0.728–0.852) in internal validation, and 0.783 (0.718–0.848) in temporal validation. In the temporal validation cohort the integrated calibration index was 0.041 at 12 months. Decision curve analysis demonstrated net clinical benefit across threshold probabilities of 10%–85%. An exploratory CKM metabolic burden score stratified 24-month event rates at 21.6%, 41.3%, and 62.5%.

**Conclusions:**

An eight-variable nomogram integrating HbA1c with CKM-axis biomarkers and diastolic function parameters predicted the long-term composite of QoL deterioration and unplanned heart failure rehospitalisation in elderly CKM stage 4a HFpEF patients, with performance preserved in temporal validation. Prospective multicentre validation in geographically distinct populations is warranted.

## 1 Introduction

Heart failure (HF) affects approximately 64 million individuals worldwide, with HF with preserved ejection fraction (HFpEF; left ventricular ejection fraction [LVEF] ≥50%) now accounting for approximately half of all HF cases and predominating in adults aged ≥70 years (1, 2). The 2024 American Heart Association Heart Disease and Stroke Statistics Update reported that HFpEF contributes substantially to the more than 6 million US adults living with HF, and that 5-year all-cause mortality in HFpEF approaches 40%, similar to HF with reduced ejection fraction despite more limited guideline-directed therapeutic options (2, 3). Approved disease-modifying interventions for HFpEF currently include sodium-glucose cotransporter-2 inhibitors (SGLT2i), glucagon-like peptide-1 receptor agonists (GLP-1 RA) in patients with obesity, and, most recently, finerenone (3).

The 2023 American Heart Association Presidential Advisory on cardiovascular-kidney-metabolic (CKM) syndrome provides a staging framework in which older HFpEF patients with metabolic risk factors and/or chronic kidney disease, but without kidney failure requiring dialysis, are classified as CKM stage 4a (4). Within this framework, cardiometabolic HFpEF has been formalised as a distinct endotype in which visceral adiposity and insulin resistance drive systemic inflammation, coronary microvascular endothelial dysfunction, myocardial fibrosis, and diastolic stiffening (5–7). Cumulative glycaemic exposure, reflected by glycated haemoglobin A1c (HbA1c), contributes to diastolic dysfunction through advanced glycation end-product formation, titin hypophosphorylation, and renal tubulointerstitial injury (5, 6).

Despite this mechanistic centrality of glycaemic metabolism, existing HFpEF risk prediction tools have not incorporated HbA1c as a continuous predictor alongside echocardiographic diastolic function parameters within a CKM-anchored architecture. The PREDICT-HFpEF model, derived from the DELIVER trial (n = 6,263; 1-year C-statistic 0.73), includes 11 clinical variables but omits HbA1c, E/e′ ratio, and left atrial volume index (LAVI) (8). A recent LASSO-based nomogram for HFpEF rehospitalisation (n = 1,149) selected the triglyceride-glucose index and E/e′ but did not retain HbA1c or LAVI (9). The MAGGIC score, derived predominantly from HF with reduced ejection fraction populations, shows a C-statistic near 0.60 when applied to HFpEF cohorts (10). Recent machine-learning models based on electronic health records have reported areas under the curve of 0.78–0.83 for HFpEF mortality but have not targeted the CKM stage 4a population (11).

In HFpEF, LVEF provides limited prognostic information because inter-patient variability is restricted by the diagnostic threshold; E/e′ ratio and LAVI instead demonstrate near-linear associations with HF hospitalisation and death, capturing instantaneous and chronic diastolic dysfunction, respectively (12). Quality-of-life (QoL) deterioration has been adopted as a co-primary endpoint in contemporary HFpEF trials, including STEP-HFpEF (semaglutide) and FINEARTS-HF (finerenone) (13, 14), and a Minnesota Living with Heart Failure Questionnaire (MLHFQ) increase of ≥5 points is the established minimal clinically important difference for this instrument (15). No existing prediction model in elderly CKM stage 4a HFpEF has targeted QoL trajectory as the primary endpoint.

We therefore aimed to develop, internally validate, and temporally validate a LASSO-Cox nomogram integrating CKM-axis biomarkers, glycaemic metabolism indicators, and echocardiographic diastolic function parameters for predicting long-term composite endpoint (quality-of-life deterioration or unplanned heart failure rehospitalisation) in elderly CKM stage 4a HFpEF patients, and to construct an exploratory metabolic burden score for within-stage risk stratification.

## 2 Materials and methods

### 2.1 Study design and ethical approval

This investigation was a single-centre retrospective observational cohort study conducted at the First Hospital of Zhangjiakou, a tertiary-care institution in Hebei Province, China. The study was approved by the Institutional Ethics Committee of the First Hospital of Zhangjiakou (reference 2024-KY-15), and the requirement for individual informed consent was waived by the Institutional Ethics Committee given the retrospective design and the use of de-identified data. The study was performed in accordance with the Declaration of Helsinki (2013 revision) and is reported following the TRIPOD+AI (Transparent Reporting of a multivariable prediction model for Individual Prognosis Or Diagnosis — Artificial Intelligence extension) 2024 checklist, which supersedes the TRIPOD 2015 checklist (16); the completed checklist is provided as **Supplementary Material**.

### 2.2 Participants

#### 2.2.1 Development cohort eligibility

The source population comprised consecutive patients aged ≥60 years admitted to the Department of Cardiology or the Department of Geriatrics between January 1, 2020, and December 31, 2023. Patients were eligible if they fulfilled all of the following: (i) a confirmed diagnosis of heart failure with preserved ejection fraction (HFpEF), defined as left ventricular ejection fraction (LVEF) ≥50% together with elevated natriuretic peptides (NT-proBNP ≥125 pg/mL for sinus rhythm or ≥365 pg/mL for atrial fibrillation) and at least one objective marker of diastolic dysfunction (E/e′ ratio >9 or left atrial volume index [LAVI] >34 mL/m²), in accordance with the 2021 European Society of Cardiology Heart Failure Guidelines (17); (ii) New York Heart Association (NYHA) functional class II–IV; (iii) comprehensive transthoracic echocardiography completed within 48 hours of index admission; and (iv) availability of a complete panel of cardiovascular-kidney-metabolic (CKM) axis laboratory biomarkers. Exclusion criteria were: (i) LVEF <50%; (ii) end-stage kidney disease on maintenance dialysis, ensuring that all participants satisfied the definition of CKM stage 4a rather than 4b; (iii) haemodynamically significant primary valvular heart disease requiring intervention; (iv) acute myocardial infarction or cardiogenic shock requiring mechanical circulatory support within the preceding 30 days; (v) active malignancy with anticipated survival <12 months; or (vi) absence of any post-discharge follow-up data. All eligible patients were symptomatic (NYHA functional class II–IV); the individual symptoms reported in **Table 1** (exertional dyspnoea, peripheral oedema, fatigue) reflect the prevalence of each specific symptom and were not individually required for eligibility.

**Table 1:** Baseline characteristics of the development cohort, overall and by training–internal validation split.

Variable	Overall (n = 302)	Training (n = 211)	Internal validation (n = 91)	P value
Demographics				
Age, years	76.2 ± 6.8	75.8 ± 6.7	77.2 ± 7.1	0.089
Female, n (%)	172 (57.0)	118 (55.9)	54 (59.3)	0.593
Height, cm	158.2 ± 8.6	158.4 ± 8.4	157.8 ± 9.0	0.582
Weight, kg	68.5 ± 12.8	68.2 ± 12.4	69.3 ± 13.6	0.493
BMI, kg/m²	27.4 ± 4.2	27.2 ± 4.1	27.8 ± 4.4	0.273
Current or former smoker, n (%)	106 (35.1)	73 (34.6)	33 (36.3)	0.779
Vital signs at admission				
Systolic BP, mmHg	138 ± 18	137 ± 17	140 ± 19	0.185
Diastolic BP, mmHg	82 ± 12	82 ± 11	82 ± 13	1.000
Heart rate, beats/min	78 ± 14	77 ± 13	80 ± 15	0.081
NYHA class III–IV, n (%)	168 (55.6)	115 (54.5)	53 (58.2)	0.553
Typical HF symptoms, n (%)				
Exertional dyspnoea	256 (84.8)	178 (84.4)	78 (85.7)	0.764
Peripheral oedema	186 (61.6)	131 (62.1)	55 (60.4%	0.787
Fatigue	222 (73.5)	154 (73.0)	68 (74.7)	0.753
Medical history, n (%)				
Hypertension	254 (84.1)	177 (83.9)	77 (84.6)	0.878
Type 2 diabetes mellitus	142 (47.0)	97 (46.0)	45 (49.5)	0.575
Atrial fibrillation or flutter	106 (35.1)	74 (35.1)	32 (35.2)	0.991
Coronary artery disease	146 (48.3)	100 (47.4)	46 (50.5)	0.614
Prior myocardial infarction	54 (17.9)	37 (17.5)	17 (18.7)	0.807
Prior stroke or TIA	68 (22.5)	46 (21.8)	22 (24.2)	0.645
COPD	46 (15.2)	32 (15.2)	14 (15.4)	0.962
Obstructive sleep apnoea	84 (27.8)	60 (28.4)	24 (26.4)	0.713
Hyperlipidaemia	158 (52.3)	108 (51.2)	50 (54.9)	0.548
CKD (eGFR <60 mL/min/1.73 m²)	128 (42.4)	86 (40.8)	42 (46.2)	0.378
Cardiovascular axis biomarkers				
NT-proBNP, pg/mL, median (IQR)	984 (430, 2050)	961 (422, 2012)	1023 (450, 2180)	0.612
ln-NT-proBNP	6.89 ± 1.24	6.87 ± 1.21	6.92 ± 1.30	0.749
hs-Troponin T, ng/L, median (IQR)	22 (14, 38)	21 (13, 36)	24 (15, 42)	0.318
Renal axis biomarkers				
Serum creatinine, μmol/L	92 ± 32	91 ± 31	95 ± 34	0.308
eGFR, mL/min/1.73 m²	62.4 ± 20.8	63.2 ± 20.4	60.6 ± 21.7	0.322
Blood urea nitrogen, mmol/L	8.6 ± 3.4	8.5 ± 3.3	8.9 ± 3.6	0.343
Serum uric acid, μmol/L	382 ± 112	378 ± 109	391 ± 118	0.365
Metabolic axis biomarkers				
Fasting blood glucose, mmol/L	6.8 ± 2.2	6.8 ± 2.1	7.0 ± 2.4	0.478
HbA1c, %	7.0 ± 1.4	7.0 ± 1.3	7.1 ± 1.5	0.564
Triglycerides, mmol/L	1.62 ± 0.84	1.60 ± 0.82	1.66 ± 0.88	0.579
Total cholesterol, mmol/L	4.36 ± 1.18	4.34 ± 1.16	4.42 ± 1.22	0.586
LDL cholesterol, mmol/L	2.58 ± 0.92	2.56 ± 0.90	2.62 ± 0.96	0.607
HDL cholesterol, mmol/L	1.12 ± 0.34	1.13 ± 0.34	1.10 ± 0.34	0.484
TyG index	8.62 ± 0.72	8.60 ± 0.70	8.66 ± 0.76	0.497
Hemoglobin glycation index (HGI)	0.14 ± 0.80	0.12 ± 0.78	0.18 ± 0.84	0.558
General laboratory values				
Hemoglobin, g/L	128 ± 20	129 ± 19	126 ± 22	0.224
White blood cell count, ×10^9^/L	7.2 ± 2.4	7.1 ± 2.3	7.4 ± 2.6	0.327
Platelet count, ×10^9^/L	198 ± 68	200 ± 67	194 ± 70	0.482
Serum albumin, g/L	38.4 ± 4.8	38.6 ± 4.7	37.9 ± 5.0	0.244
hs-CRP, mg/L, median (IQR)	4.8 (1.8, 11.6)	4.6 (1.7, 11.2)	5.2 (2.0, 12.4)	0.434
Echocardiography				
LVEF, %	59.4 ± 5.8	59.6 ± 5.6	58.9 ± 6.1	0.338
LVEDD, mm	48.2 ± 5.6	48.0 ± 5.5	48.6 ± 5.8	0.396
LVESD, mm	31.4 ± 4.8	31.2 ± 4.7	31.8 ± 5.0	0.321
Interventricular septum thickness, mm	11.2 ± 1.8	11.1 ± 1.8	11.4 ± 1.9	0.192
Posterior wall thickness, mm	10.8 ± 1.6	10.7 ± 1.6	11.0 ± 1.7	0.149
LV mass index, g/m²	118 ± 32	117 ± 31	121 ± 34	0.316
LAVI, mL/m²	45.6 ± 12.8	45.1 ± 12.3	46.7 ± 13.8	0.304
E/A ratio	1.08 ± 0.42	1.06 ± 0.40	1.12 ± 0.46	0.248
E/e′ ratio (average)	16.8 ± 5.4	16.5 ± 5.1	17.3 ± 5.9	0.229
TR velocity, m/s	2.84 ± 0.54	2.82 ± 0.52	2.88 ± 0.58	0.367
TAPSE, mm	19.4 ± 3.6	19.5 ± 3.5	19.2 ± 3.8	0.488
Baseline quality of life				
MLHFQ total score (0–105)	35.0 ± 14.6	34.6 ± 14.2	35.9 ± 15.4	0.466
Medications at discharge, n (%)				
ACEI or ARB	184 (60.9)	128 (60.7)	56 (61.5)	0.887
ARNI (sacubitril-valsartan)	54 (17.9)	38 (18.0)	16 (17.6)	0.928
Beta-blocker	224 (74.2)	156 (73.9)	68 (74.7)	0.886
MRA (spironolactone or eplerenone)	170 (56.3)	118 (55.9)	52 (57.1)	0.846
SGLT2 inhibitor	118 (39.1)	83 (39.3)	35 (38.5)	0.889
GLP-1 receptor agonist	24 (7.9)	16 (7.6)	8 (8.8)	0.723
Finerenone	24 (7.9)	17 (8.1)	7 (7.7)	0.914
Loop diuretic	262 (86.8)	182 (86.3)	80 (87.9)	0.698
Thiazide or thiazide-like diuretic	42 (13.9)	30 (14.2)	12 (13.2)	0.811
Statin	206 (68.2)	144 (68.2)	62 (68.1)	0.986
Antiplatelet agent	126 (41.7)	88 (41.7)	38 (41.8)	0.992
Oral anticoagulant	94 (31.1)	65 (30.8)	29 (31.9)	0.855
Digoxin	36 (11.9)	25 (11.8)	11 (12.1)	0.951
≥3 guideline-directed classes	216 (71.5)	152 (72.0)	64 (70.3)	0.760

Data are presented as mean ± SD, median (IQR), or n (%). P values from Student’s t-test, Mann–Whitney U test, or Pearson χ² test comparing training vs internal validation. BMI, body mass index; BP, blood pressure; NYHA, New York Heart Association; TIA, transient ischemic attack; COPD, chronic obstructive pulmonary disease; CKD, chronic kidney disease; eGFR, estimated glomerular filtration rate (CKD-EPI 2021); NT-proBNP, N-terminal pro-B-type natriuretic peptide; hs, high-sensitivity; HbA1c, glycated haemoglobin A1c; TyG, triglyceride-glucose index; HGI, haemoglobin glycation index; LDL, low-density lipoprotein; HDL, high-density lipoprotein; CRP, C-reactive protein; LVEF, left ventricular ejection fraction; LVEDD, LV end-diastolic dimension; LVESD, LV end-systolic dimension; LAVI, left atrial volume index; TR, tricuspid regurgitation; TAPSE, tricuspid annular plane systolic excursion; MLHFQ, Minnesota Living with Heart Failure Questionnaire; ACEI, angiotensin-converting enzyme inhibitor; ARB, angiotensin receptor blocker; ARNI, angiotensin receptor–neprilysin inhibitor; MRA, mineralocorticoid receptor antagonist; SGLT2, sodium-glucose cotransporter-2; GLP-1, glucagon-like peptide-1.

#### 2.2.2 Temporal validation cohort

A temporal validation cohort was assembled from consecutive patients admitted to the same departments between January 1, 2024, and June 30, 2025, applying identical eligibility criteria, identical HFpEF diagnostic standards, identical CKM stage 4a classification rules, and identical baseline sampling conventions. This cohort was entirely non-overlapping with the development cohort in both time and patient identity.

#### 2.2.3 Sample size considerations

The minimum sample size for the development cohort was determined using the criteria of Riley and colleagues for multivariable prediction models (18). Assuming a target C-index of 0.80, a composite endpoint prevalence of approximately 38%, and a maximum of eight predictor parameters in the final model, the calculated minimum was 288 patients to achieve a shrinkage factor ≥0.9 and a mean absolute prediction error ≤0.05. The available cohort of 302 patients exceeded this threshold.

### 2.3 Baseline data collection and predictor variables

#### 2.3.1 Sampling timing

All baseline laboratory biomarkers were obtained from the first routine laboratory panel drawn within 24 hours of hospital admission, prior to initiation of acute decongestive or glucose-lowering therapy. Samples obtained during active acute coronary syndrome, documented acute infection (C-reactive protein >50 mg/L with clinical suspicion of active infection), or haemodynamic instability requiring inotropic or vasopressor support were not used as analytic baseline; for such patients, the baseline panel was re-drawn on the first clinically stable day.

#### 2.3.2 CKM cardiovascular axis

Plasma N-terminal pro-B-type natriuretic peptide (NT-proBNP) was quantified by electrochemiluminescence immunoassay (Cobas e601, Roche Diagnostics, Mannheim, Germany). Because of right skew (skewness >2.0), a natural logarithmic transformation (ln-NT-proBNP) was applied before modelling.

#### 2.3.3 CKM renal axis

Serum creatinine was measured enzymatically on the Cobas c702 platform (Roche Diagnostics). The estimated glomerular filtration rate (eGFR) was calculated using the 2021 Chronic Kidney Disease Epidemiology Collaboration (CKD-EPI) creatinine equation without a race coefficient, as recommended by the National Kidney Foundation–American Society of Nephrology Task Force (19). Serum uric acid was determined by the uricase–peroxidase colorimetric method.

#### 2.3.4 CKM metabolic axis

Glycated haemoglobin A1c (HbA1c) was measured by ion-exchange high-performance liquid chromatography (Bio-Rad D-10 Hemoglobin Analyzer). Fasting blood glucose (FBG) was measured after an overnight fast of ≥8 hours. The triglyceride-glucose (TyG) index was calculated as ln[fasting triglycerides (mg/dL) × FBG (mg/dL)/2]. The haemoglobin glycation index (HGI) was calculated as the residual from a cohort-level ordinary least-squares regression of HbA1c on FBG (HGI = observed HbA1c − predicted HbA1c). Serum triglycerides and low-density lipoprotein cholesterol were measured by standard enzymatic assays.

#### 2.3.5 Echocardiography

Standardised comprehensive transthoracic echocardiography was performed within 48 hours of admission using a Philips EPIQ 7C or GE Vivid E95 ultrasound system (phased-array transducer, 2.5–3.5 MHz). Studies were acquired and interpreted by two board-certified cardiovascular imaging specialists (each with ≥10 years of experience) who were blinded to clinical, laboratory, and outcome data, following the 2015 American Society of Echocardiography–European Association of Cardiovascular Imaging (ASE–EACVI) recommendations for cardiac chamber quantification (20). LVEF was measured by the biplane Simpson method. LAVI was measured by the biplane area–length method indexed to body surface area. E/e′ ratio was calculated as the ratio of peak early transmitral inflow velocity to the average of septal and lateral early diastolic mitral annular tissue Doppler velocities; in patients with atrial fibrillation, the average of ≥5 consecutive cardiac cycles was used.

#### 2.3.6 CKM stage 4a metabolic burden score

An exploratory binary equal-weight composite score ranging from 0 to 6 points was constructed, with each item anchored to an externally recommended threshold: (i) eGFR <60 mL/min/1.73 m²; (ii) HbA1c >7.0%; (iii) uric acid >450 μmol/L; (iv) body mass index ≥30 kg/m²; (v) low-density lipoprotein cholesterol >2.6 mmol/L; and (vi) TyG index ≥8.5. Scores were trichotomised as low (0–1), intermediate (2–3), and high (4–6) burden.

### 2.4 Outcome definition and follow-up

Follow-up commenced on the date of the baseline echocardiographic examination and was censored at the earliest of (a) occurrence of the primary composite endpoint, (b) all-cause mortality treated as a competing event, (c) the pre-specified data lock date of June 30, 2025, or (d) last contact for patients lost to follow-up. The Minnesota Living with Heart Failure Questionnaire (MLHFQ) was administered at baseline and at each subsequent annual outpatient visit (approximately 12 and 24 months post-enrolment) by dedicated specialist nurses. The primary composite endpoint was the time from baseline to whichever of the following occurred first: (a) clinically meaningful quality-of-life deterioration, defined as an MLHFQ total score increase ≥5 points from baseline, or (b) unplanned heart failure rehospitalisation, defined as any non-elective admission with a primary discharge diagnosis of heart failure. Pre-specified sensitivity analyses used alternative MLHFQ thresholds of ≥3 points (more sensitive), ≥7 points (more specific), and a ≥10% relative-change definition that accounts for baseline MLHFQ heterogeneity.

### 2.5 Statistical analysis

#### 2.5.1 Missing data handling

Incomplete observations were addressed using multiple imputation by chained equations (MICE; mice R package v3.16.0), generating m = 5 imputed datasets with 20 iterations per chain. The imputation model included all 35 candidate predictors, the Nelson–Aalen cumulative hazard estimate, the natural log of follow-up time, and the binary event indicator. Continuous variables were imputed using predictive mean matching, and binary variables using logistic regression. For cohort eligibility, a ‘complete CKM-axis panel’ was strictly defined as the presence of baseline eGFR, serum uric acid, and NT-proBNP, which are essential for the baseline diagnosis of HFpEF and the classification of CKM Stage 4a. While HbA1c is a central metabolic marker, its absence does not preclude a CKM Stage 4a diagnosis (which can be satisfied by CKD or obesity alone). To prevent severe selection bias, patients with missing HbA1c (17.6%) were retained, and missing values were handled using Multiple Imputation by Chained Equations (MICE). Continuous variables were imputed using Predictive Mean Matching (PMM), which preserves non-linear distributions by borrowing observed values from donor patients with the closest predicted regression scores. Binary variables were imputed using multiple logistic regression.

#### 2.5.2 Nonlinear dose–response analyses

Restricted cubic spline (RCS) Cox regression with four knots placed at the 5th, 35th, 65th, and 95th percentiles of each predictor distribution was used to characterise potential nonlinear associations between six *a priori* continuous predictors (uric acid, ln-NT-proBNP, eGFR, HbA1c, TyG index, HGI) and the hazard of the composite endpoint. Each RCS plot reports the Wald chi-square P-value for overall association (P-overall) and for departure from linearity (P-nonlinear).

#### 2.5.3 Feature selection by LASSO-Cox regression

All 35 candidate predictors were z-score standardised and entered into a LASSO-penalised Cox proportional hazards model. To integrate multiple imputation with LASSO, a three-stage procedure was adopted: (i) LASSO-Cox was fitted independently on each of the five imputed datasets using 10-fold cross-validation, with both the minimum-deviance model (λ.min) and the one-standard-error model (λ.1se) examined; (ii) the final predictor set was defined as variables retaining non-zero coefficients across all five datasets (selection frequency 5/5 = 100%); and (iii) an unpenalised Cox model was fitted on each imputed dataset using only the final predictor set, with point estimates, standard errors, and variance–covariance matrices pooled on the log hazard ratio scale using Rubin’s rules. The λ.min model served as the primary analysis. The dataset was randomly partitioned into a training set (70%) and an internal validation set (30%). This split was formally stratified by the primary composite event status to ensure equivalent event rates across both cohorts. To prevent data leakage, LASSO feature selection and bootstrap optimism correction were performed exclusively on the training set. We deliberately employed LASSO rather than fitting an unpenalised Cox model on all 35 candidate predictors, as the latter approach would result in an unacceptable Events-Per-Variable (EPV) ratio, severely violating statistical assumptions and leading to model overfitting. To explain the model output, SHapley Additive exPlanations (SHAP) values were computed on the log-hazard ratio scale (the linear predictor) of the final Cox model using the fastshap and survival packages.

#### 2.5.4 Nomogram construction and incremental predictive value

A graphical nomogram was constructed from the pooled multivariable Cox regression coefficients to estimate 12-month and 24-month probabilities of the primary composite endpoint. A SHapley Additive exPlanations (SHAP) summary plot was generated to visualise the direction and relative magnitude of each predictor’s contribution. To quantify the incremental value of each predictor domain, four sequentially nested Cox models were compared: Model 1 (age + NYHA class + resting heart rate), Model 2 (+ ln-NT-proBNP + uric acid), Model 3 (+ E/e′ + LAVI), and Model 4 (+ HbA1c). For each step, change in Harrell’s C-index, continuous and category-based net reclassification improvement (NRI), integrated discrimination improvement (IDI), and the likelihood ratio test were computed. The proportional hazards assumption for each retained predictor was verified using Schoenfeld residual tests; no variable demonstrated a statistically significant departure from proportionality (all *P* > 0.10).

#### 2.5.5 Model validation

Discrimination was assessed by Harrell’s C-index and by time-dependent receiver operating characteristic curves at 12 and 24 months. Calibration was evaluated using a hierarchy of metrics: calibration-in-the-large intercept, calibration slope, integrated calibration index (ICI), expected-to-observed ratio, and 1,000-bootstrap resampled calibration plots. Internal validation used 1,000-iteration bootstrap resampling of the full modelling pipeline, in which the LASSO variable-selection step was re-executed within each bootstrap sample, to derive optimism-corrected discrimination and calibration. The final eight-variable model derived in the development cohort was applied without recalibration to the temporal validation cohort, and the same discrimination and calibration metrics were computed. Although current best practice for prediction model development in moderate-sized cohorts recommends using the full cohort with bootstrap internal validation rather than data splitting (Riley 2020), we retained the 70:30 split to provide complementary cross-validation within the development cohort, with bootstrap optimism correction applied to the full modelling pipeline on the training set. The temporal validation cohort (n = 102) provides the principal out-of-sample validation.

#### 2.5.6 Clinical utility assessment

Clinical utility was assessed using decision curve analysis across three pre-specified clinical decision scenarios: (i) intensified outpatient follow-up (threshold probability 15–25%); (ii) initiation of CKM-directed pharmacotherapy including SGLT2 inhibitor or GLP-1 receptor agonist (30–45%); and (iii) multidisciplinary CKM referral (50–65%). Net benefit of the nomogram was compared with treat-all and treat-none reference strategies within each scenario. Net benefit was calculated according to the method of Vickers and Elkin as net benefit = (TP/N) − (FP/N) × [p_t_/(1 − p_t_)], where N is the total number of patients; TP and FP are, respectively, the numbers of patients with and without the primary composite endpoint amongst those classified as high-risk at a given threshold probability p_t_; and p_t_ is the threshold probability at which a patient or clinician would opt for the intervention under consideration. The factor p_t_/(1 − p_t_) represents the exchange rate between the harm of an unnecessary intervention (a false positive) and the harm of a missed event (a false negative). The range of threshold probabilities examined (10–85%) corresponds to the union of the three pre-specified decision scenarios — intensified outpatient follow-up (≈15–25%), CKM-directed pharmacotherapy (≈30–45%), and multidisciplinary CKM referral (≈50–65%) — with modest margins, bounded below by the risk at which a low-burden intervention becomes reasonable and above by the risk at which only patients at very high probability of the endpoint would accept the most resource-intensive pathway; above approximately 85% essentially no patient was classified as high-risk.

#### 2.5.7 Competing risk and sensitivity analyses

Because all-cause mortality precludes observation of the primary endpoint, a Fine–Gray subdistribution hazard model was fitted with all-cause mortality as the competing event, and subdistribution hazard ratios were compared against cause-specific hazard ratios from the primary Cox model. Aalen–Johansen cumulative incidence function curves were generated stratified by nomogram risk tertiles. A 6-month landmark analysis was performed to mitigate immortal time bias. Subgroup analyses with interaction tests were performed across six pre-specified strata: sex, age (<75 vs ≥75 years), diabetes status, chronic kidney disease status (eGFR <60 vs ≥60 mL/min/1.73 m²), NYHA class (II vs III–IV), and intensity of guideline-directed medical therapy (≥3 vs <3 drug classes at discharge). All statistical analyses were performed using R software v4.3.1 (R Foundation for Statistical Computing, Vienna, Austria), with primary packages mice, glmnet, rms, survival, survminer, timeROC, and cmprsk. Two-sided P-values <0.05 were considered statistically significant.

#### 2.5.8 Model validation and clinical utility

Furthermore, to facilitate practical clinical decision-making, the optimal probability cut-off value for predicting the 24-month primary composite endpoint was determined by maximising the Youden index (Sensitivity + Specificity − 1) derived from the time-dependent ROC curve. Based on this optimal threshold, the corresponding sensitivity, specificity, positive predictive value (PPV), and negative predictive value (NPV) were systematically calculated. Additionally, to verify its independent predictive ability and incremental value, the predictive performance (AUC) of our model was benchmarked against the traditional MAGGIC (Meta-Analysis Global Group in Chronic Heart Failure) prognostic risk score using DeLong’s test.

## 3 Results

### 3.1 Study population and follow-up

**Figure 1:**
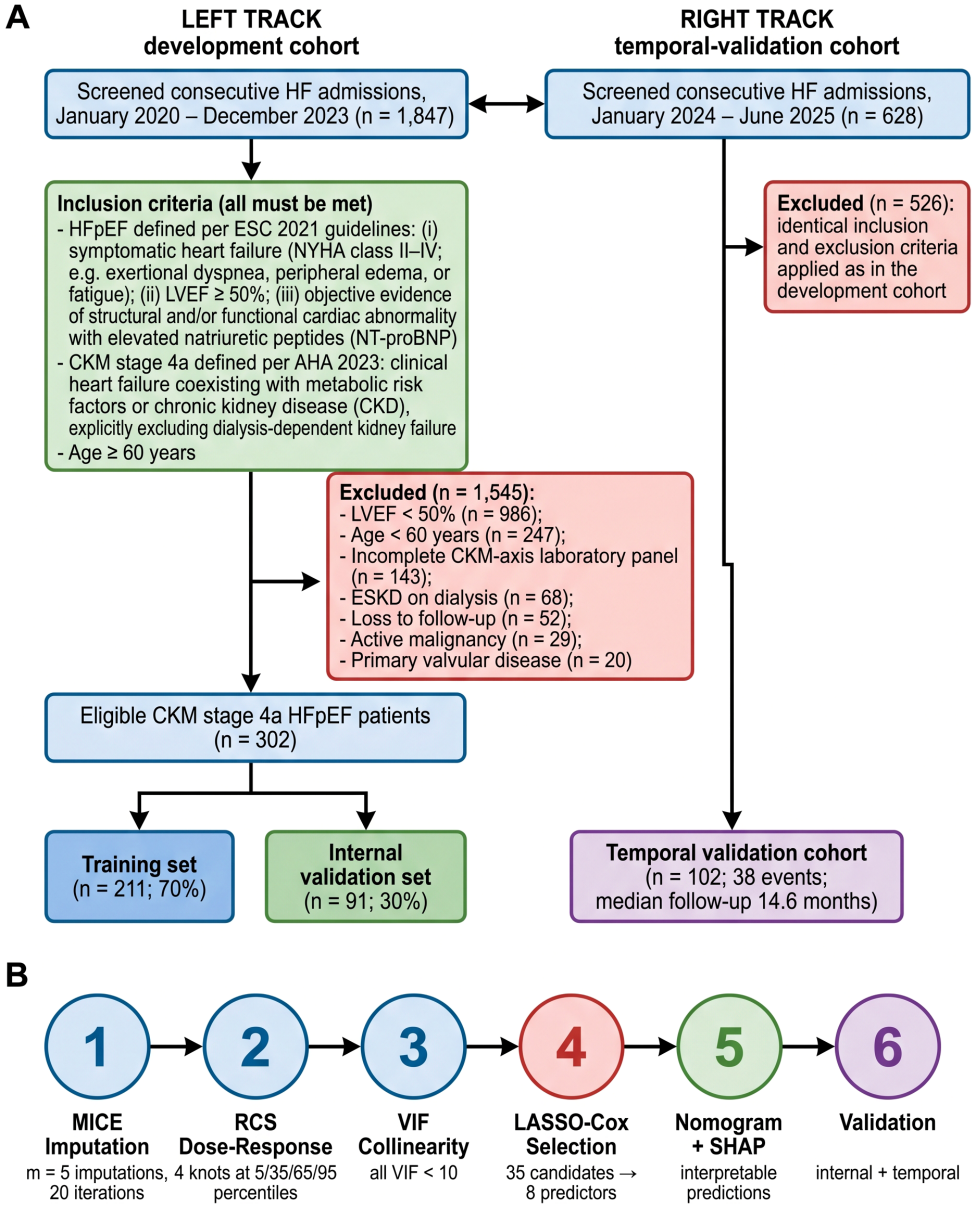
Study flowchart and analytic pipeline. Patient selection flowchart: 1,847 screened → 302 enrolled in the development cohort (training 211 + internal validation 91), with CONSORT-style accounting of exclusions. A parallel temporal validation cohort (n = 102) assembled under identical criteria from consecutive admissions between January 2024 and June 2025 is shown alongside.

Between January 1, 2020 and December 31, 2023, 1,847 patients with a primary discharge diagnosis of heart failure were screened at the First Hospital of Zhangjiakou. After sequential application of eligibility criteria, 302 patients with confirmed HFpEF meeting CKM stage 4a criteria were enrolled in the development cohort (**Figure 1a**). The most common reasons for exclusion were LVEF <50% (n = 986), age <60 years (n = 247), incomplete CKM-axis laboratory panel (n = 143), end-stage kidney disease on maintenance dialysis (n = 68), and loss to follow-up (n = 52). The development cohort was partitioned into a training set (n = 211; 70%) and an internal validation set (n = 91; 30%). A separate temporal validation cohort (n = 102) was assembled from consecutive patients admitted between January 1, 2024 and June 30, 2025 applying identical eligibility criteria. Baseline characteristics of the development cohort are summarised in **Table 1**. Mean age was 76.2 ± 6.8 years and 57.0% of participants were female. NYHA functional class III–IV was present in 55.6%, hypertension in 84.1%, type 2 diabetes in 47.0%, atrial fibrillation in 35.1%, and chronic kidney disease (eGFR <60 mL/min/1.73 m²) in 42.4%. Mean HbA1c was 7.0 ± 1.4%, mean E/e′ was 16.8 ± 5.4, and mean LAVI was 45.6 ± 12.8 mL/m². No variable differed significantly between the training and internal validation sets (all P > 0.05). Median follow-up was 22.4 months (interquartile range 14.8–28.6). The primary composite endpoint occurred in 116 patients (38.4%); all-cause mortality occurred in 32 patients (10.6%) and was treated as a competing event. Cumulative incidence of the primary endpoint at 12 and 24 months was 18.5% (95% CI 14.2–23.4%) and 42.7% (95% CI 37.0–48.6%), respectively.

### 3.2 Univariable comparisons and missing data

Univariable comparisons between event and event-free groups are shown in **Table 2**. Patients who reached the primary endpoint were older (78.4 ± 6.8 vs 74.8 ± 6.5 years; P < 0.001), more frequently in NYHA class III–IV (69.0% vs 47.3%; P < 0.001), and had higher median NT-proBNP, lower eGFR, higher serum uric acid, higher E/e′ ratio (20.0 ± 5.2 vs 14.8 ± 4.6; P < 0.001), and higher LAVI (52.6 ± 12.6 vs 41.2 ± 10.8 mL/m²; P < 0.001). All three glycaemic metabolism indicators differed significantly between groups: HbA1c (7.5 ± 1.5% vs 6.6 ± 1.1%; P < 0.001), TyG index (8.88 ± 0.74 vs 8.46 ± 0.66; P < 0.001), and HGI (0.34 ± 0.88 vs 0.02 ± 0.74; P = 0.001). LVEF did not differ between groups (59.8 ± 6.7% vs 60.4 ± 6.2%; P = 0.43). Variable-specific missing rates (**Supplementary Table 1**) showed HbA1c with the highest missingness (17.6%), followed by tricuspid regurgitation velocity (15.6%) and TAPSE (9.8%); all other variables had <10% missingness. Kernel density overlay plots (**Supplementary Figure 1**) confirmed that imputed distributions closely matched the observed data for all variables (Kolmogorov–Smirnov P > 0.05 for all).

**Table 2:** Univariable comparison of baseline characteristics by primary composite endpoint status in the development cohort.

Variable	Event-free (n = 186)	Primary endpoint (n = 116)	P value
Demographics			
Age, years	74.8 ± 6.5	78.4 ± 6.8	<0.001
Female, n (%)	104 (55.9)	68 (58.6)	0.642
BMI, kg/m²	27.0 ± 4.0	28.0 ± 4.4	0.047
Current or former smoker, n (%)	62 (33.3)	44 (37.9)	0.413
Vital signs			
Systolic BP, mmHg	139 ± 17	136 ± 19	0.156
Diastolic BP, mmHg	82 ± 11	82 ± 13	1.000
Heart rate, beats/min	76 ± 13	82 ± 15	<0.001
NYHA III–IV, n (%)	88 (47.3)	80 (69.0)	<0.001
Medical history, n (%)			
Hypertension	156 (83.9)	98 (84.5)	0.889
Type 2 diabetes mellitus	82 (44.1)	60 (51.7)	0.198
Atrial fibrillation or flutter	62 (33.3)	44 (37.9)	0.413
Coronary artery disease	84 (45.2)	62 (53.4)	0.162
Prior myocardial infarction	28 (15.1)	26 (22.4)	0.102
Prior stroke or TIA	36 (19.4)	32 (27.6)	0.095
COPD	24 (12.9)	22 (19.0)	0.153
Obstructive sleep apnoea	46 (24.7)	38 (32.8)	0.129
Hyperlipidaemia	92 (49.5)	66 (56.9)	0.208
CKD (eGFR <60)	68 (36.6)	60 (51.7)	0.010
Cardiovascular axis			
NT-proBNP, pg/mL, median (IQR)	650 (318, 1385)	1876 (842, 3720)	<0.001
ln-NT-proBNP	6.48 ± 1.15	7.54 ± 1.18	<0.001
hs-Troponin T, ng/L, median (IQR)	18 (11, 30)	32 (20, 56)	<0.001
Renal axis			
Serum creatinine, μmol/L	86 ± 28	102 ± 36	<0.001
eGFR, mL/min/1.73 m²	66.8 ± 19.4	55.4 ± 21.2	<0.001
Blood urea nitrogen, mmol/L	7.9 ± 3.0	9.6 ± 3.7	<0.001
Serum uric acid, μmol/L	358 ± 102	420 ± 120	<0.001
Metabolic axis			
Fasting blood glucose, mmol/L	6.4 ± 1.8	7.4 ± 2.6	<0.001
HbA1c, %	6.6 ± 1.1	7.5 ± 1.5	<0.001
Triglycerides, mmol/L	1.54 ± 0.78	1.74 ± 0.92	0.046
Total cholesterol, mmol/L	4.30 ± 1.14	4.46 ± 1.24	0.255
LDL cholesterol, mmol/L	2.54 ± 0.88	2.64 ± 0.98	0.361
HDL cholesterol, mmol/L	1.14 ± 0.34	1.09 ± 0.34	0.213
TyG index	8.46 ± 0.66	8.88 ± 0.74	<0.001
HGI	0.02 ± 0.74	0.34 ± 0.88	0.001
General laboratory			
Hemoglobin, g/L	131 ± 19	123 ± 21	<0.001
Serum albumin, g/L	39.2 ± 4.6	37.1 ± 4.9	<0.001
hs-CRP, mg/L, median (IQR)	3.8 (1.4, 9.2)	7.2 (2.8, 16.4)	<0.001
Echocardiography			
LVEF, %	60.4 ± 6.2	59.8 ± 6.7	0.430
LVMI, g/m²	112 ± 30	128 ± 34	<0.001
LAVI, mL/m²	41.2 ± 10.8	52.6 ± 12.6	<0.001
E/A ratio	1.06 ± 0.38	1.12 ± 0.48	0.245
E/e′ ratio	14.8 ± 4.6	20.0 ± 5.2	<0.001
TR velocity, m/s	2.72 ± 0.50	3.04 ± 0.56	<0.001
TAPSE, mm	20.1 ± 3.4	18.3 ± 3.6	<0.001
Baseline MLHFQ total score	32.8 ± 14.2	38.6 ± 15.8	0.001
Medications at discharge, n (%)			
ACEI/ARB/ARNI	148 (79.6)	90 (77.6)	0.676
Beta-blocker	138 (74.2)	86 (74.1)	0.992
MRA	100 (53.8)	70 (60.3)	0.268
SGLT2 inhibitor	82 (44.1)	36 (31.0)	0.024
GLP-1 receptor agonist	14 (7.5)	10 (8.6)	0.734
Finerenone	14 (7.5)	10 (8.6)	0.734
≥3 guideline-directed classes	138 (74.2)	78 (67.2)	0.188

Data are mean ± SD, median (IQR), or n (%). P values from Student’s t-test, Mann–Whitney U test, or Pearson χ² test as appropriate. The SGLT2 inhibitor comparison (P = 0.024) reflects univariable association subject to confounding by indication; the study does not claim a causal benefit. Abbreviations as in **Table 1**. P values are descriptive and not adjusted for multiple comparisons; variable selection for the multivariable model was performed using LASSO-Cox rather than univariable screening.

### 3.3 Nonlinear dose–response relationships

**Figure 2:**
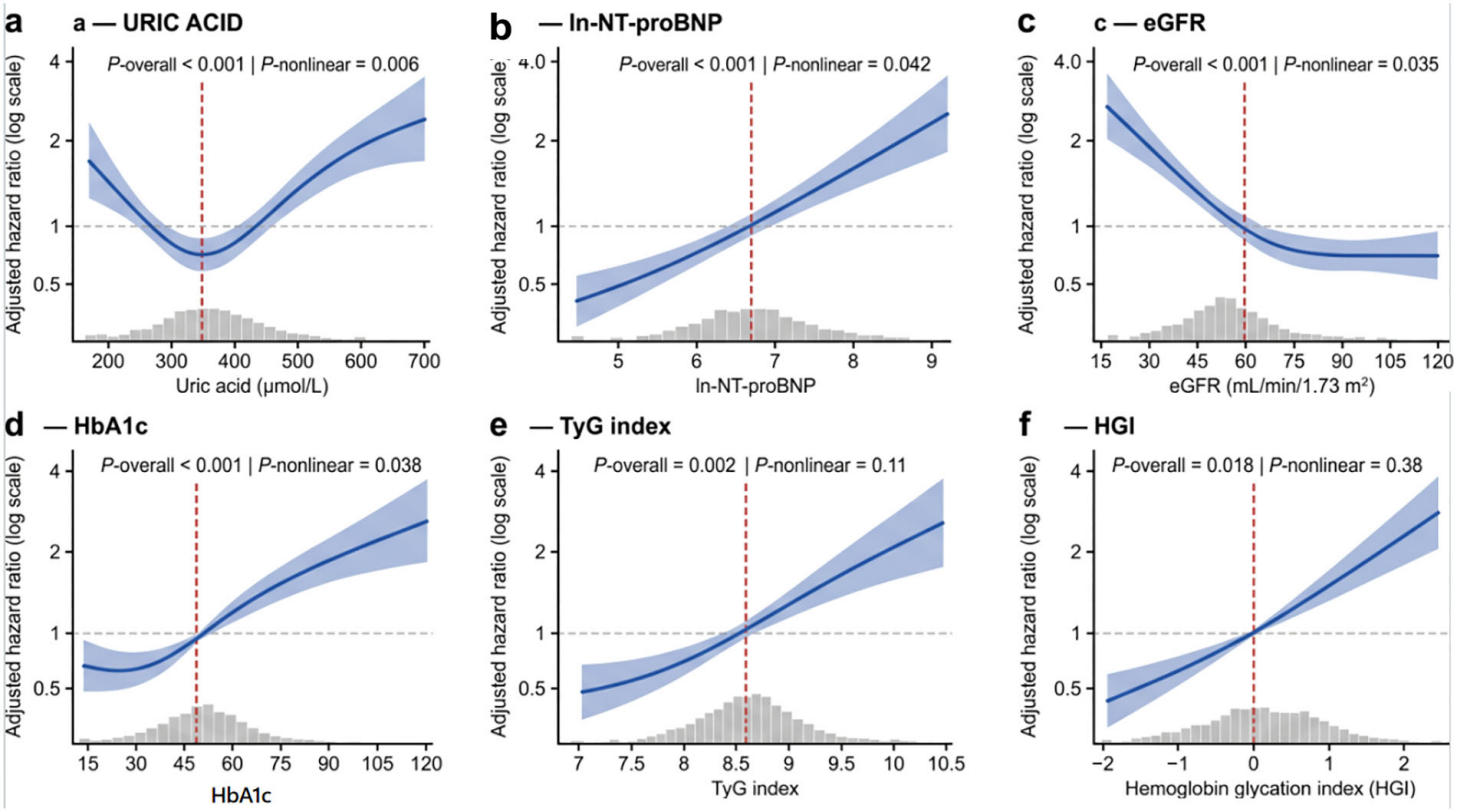
Restricted cubic spline analyses of six *a priori* continuous predictors in the training set. Adjusted hazard ratios with 95% confidence intervals (shaded bands) for the primary composite endpoint, modelled with four knots at the 5th, 35th, 65th, and 95th percentiles. Reference set at the median of each predictor. Models adjusted for age, sex, and NYHA class. **(a)** Uric acid: U-shaped relationship (P-nonlinear = 0.006), risk nadir near 350 μmol/L. **(b)** ln-NT-proBNP: J-shaped monotonically increasing curve (P-nonlinear = 0.042). **(c)** eGFR: inverse nonlinear association with plateau above 75 mL/min/1.73 m² (P-nonlinear = 0.035). **(d)** HbA1c: J-shaped relationship with risk escalating above 7.5% (P-nonlinear = 0.038). **(e)** TyG index: J-shaped curve with inflection near 9.0 (P-nonlinear = 0.11). **(f)** HGI: near-linear positive dose–response relationship (P-nonlinear = 0.38).

Restricted cubic spline analyses of six *a priori* continuous predictors are shown in **Figure 2**. Uric acid exhibited a U-shaped relationship with the composite endpoint (P-overall < 0.001; P-nonlinear = 0.006), with a risk nadir near 350 μmol/L (**Figure 2a**). Ln-NT-proBNP demonstrated a J-shaped monotonically increasing curve (P-overall < 0.001; P-nonlinear = 0.042; **Figure 2b**). eGFR displayed an inverse nonlinear association, with accelerating risk below 45 mL/min/1.73 m² and a plateau above 75 mL/min/1.73 m² (P-overall < 0.001; P-nonlinear = 0.035; **Figure 2c**). HbA1c showed a J-shaped pattern with risk increasing above 7.5% (P-overall < 0.001; P-nonlinear = 0.038; **Figure 2d**). The TyG index showed a J-shaped curve with an inflection point near 9.0 (P-overall = 0.002; P-nonlinear = 0.11; **Figure 2e**), and HGI demonstrated a near-linear positive association (P-overall = 0.018; P-nonlinear = 0.38; **Figure 2f**).

### 3.4 Collinearity and LASSO feature selection

**Figure 3:**
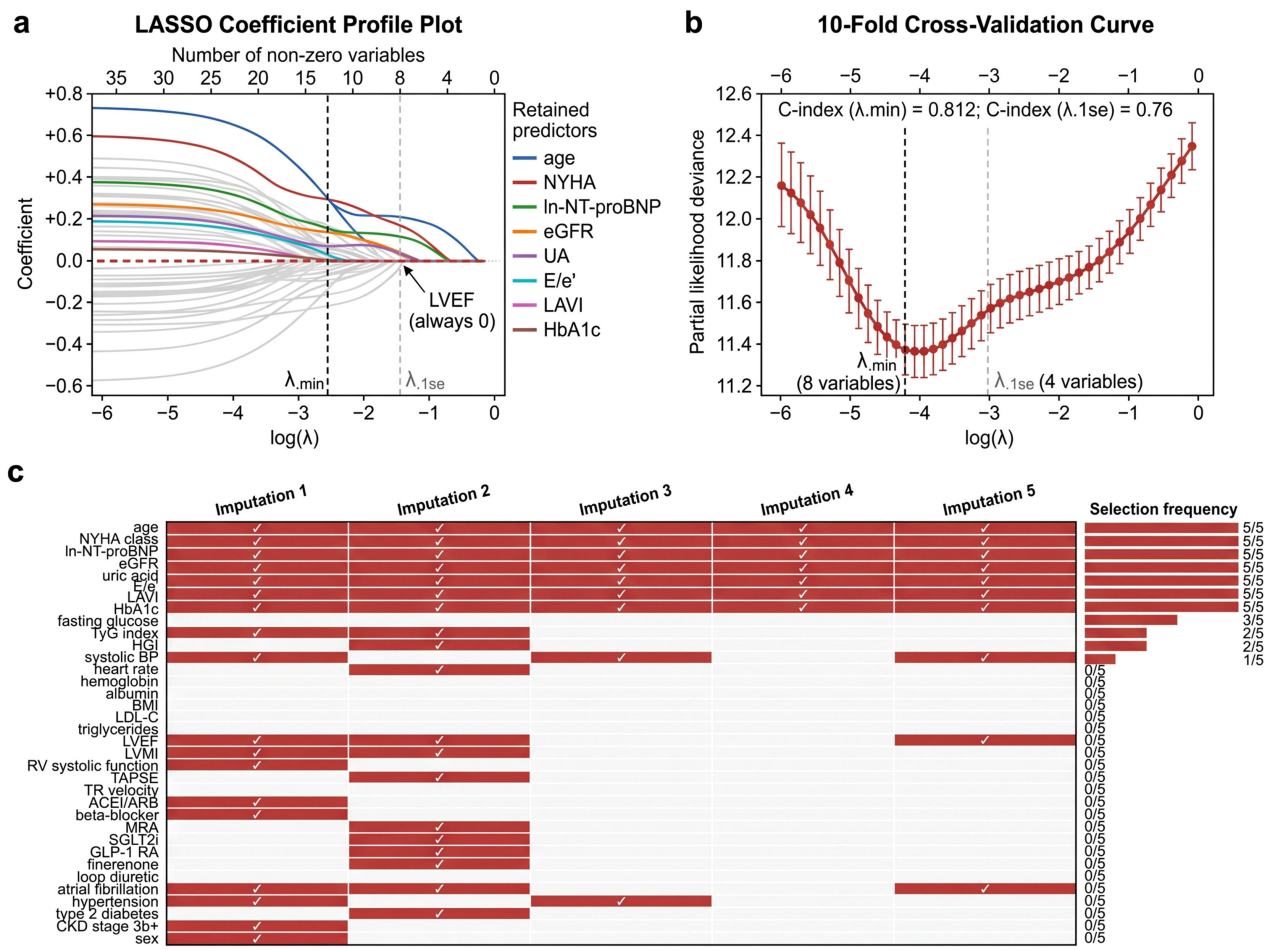
LASSO-Cox feature selection. **(a)** Coefficient profile plot showing the trajectory of each candidate variable’s coefficient as a function of log(λ); LVEF trajectory remains at zero across the entire λ path. **(b)** 10-fold cross-validation curve of partial likelihood deviance; vertical dashed lines mark λ.min (8 variables; primary model) and λ.1se (4 variables). **(c)** Selection frequency heatmap across the five multiply imputed datasets at λ.min; the eight predictors with 5/5 selection form the final model.

Variance inflation factors (VIFs) for all 35 candidate predictors were below 10; the highest VIF was observed between left ventricular end-diastolic dimension and LAVI (VIF = 4.2). LVEF had a VIF of 1.8, reflecting its restricted range in this pure HFpEF cohort. The LASSO coefficient path and 10-fold cross-validation curve are presented in **Figures 3a, b**. At the minimum-deviance regularisation parameter (λ.min), eight variables retained non-zero coefficients across all five imputed datasets: age, NYHA class, ln-NT-proBNP, eGFR, uric acid, E/e′ ratio, LAVI, and HbA1c (**Figure 3c**; **Supplementary Figure 2**). LVEF was compressed to zero in all five imputed datasets; fasting blood glucose, TyG index, HGI, systolic blood pressure, haemoglobin, albumin, and all medication variables were similarly penalised to zero. After adjustment for the other LASSO-selected variables, HbA1c retained moderate-to-strong partial correlations with TyG (r = 0.58; P < 0.001) and HGI (r = 0.63; P < 0.001), consistent with informational absorption by HbA1c. The more parsimonious λ.1se model retained only four variables (ln-NT-proBNP, E/e′, LAVI, HbA1c) with a C-index of 0.76, compared with 0.81 for λ.min, supporting use of the λ.min model as the primary analysis.

### 3.5 Multivariable Cox model and nomogram

**Figure 4:**
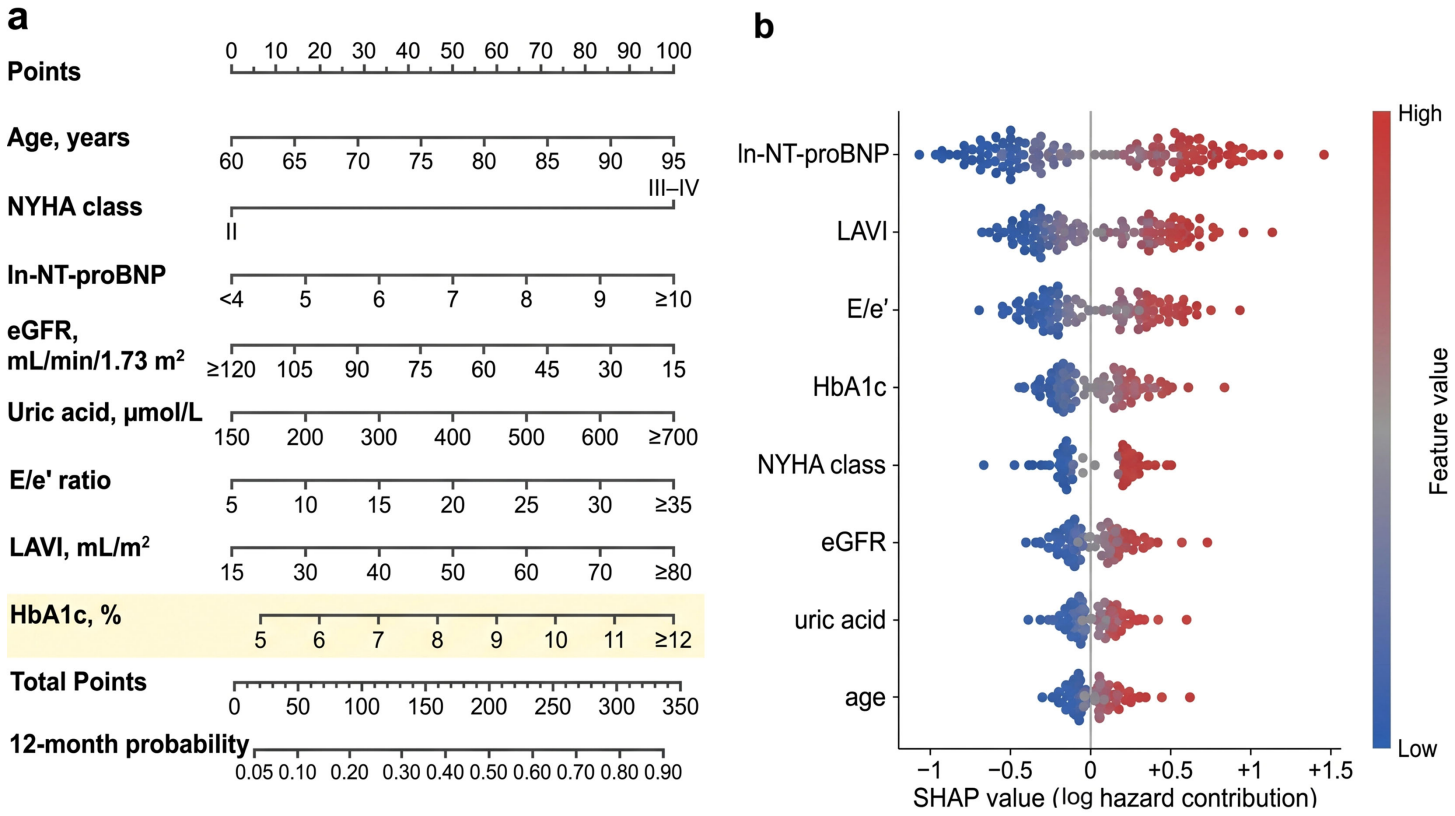
Nomogram and SHAP summary plot. **(a)** Cox regression nomogram constructed from the eight LASSO-selected predictors; each predictor axis is scaled to a common 0–100 “Points” scale at the top. The HbA1c axis is highlighted to indicate its role as the novel glycaemic predictor. The “Total Points” sum is mapped to 12-month and 24-month probabilities of the primary composite endpoint via the two bottom scales. **(b)** SHapley Additive exPlanations beeswarm summary plot. Each row represents a predictor sorted by descending mean absolute SHAP value; each dot represents an individual patient coloured by feature value (blue = low, red = high).

The pooled multivariable Cox proportional hazards model of the eight LASSO-selected variables is shown in **Table 3**. All eight predictors demonstrated statistically significant independent associations with the composite endpoint. The nomogram derived from these eight predictors is shown in **Figure 4a**, providing graphical estimation of 12-month and 24-month probabilities of the primary composite endpoint. The SHAP summary plot (**Figure 4b**) identified ln-NT-proBNP, LAVI, E/e′, and HbA1c as the four predictors with the largest mean absolute SHAP values, with NYHA class, eGFR, uric acid, and age providing smaller but consistent contributions.

**Table 3:** Multivariable Cox regression of LASSO-selected predictors (pooled across five imputed datasets by Rubin’s rules).

Variable	β	SE	HR (95% CI)	P value
Age, per year	0.039	0.013	1.04 (1.01–1.06)	0.002
NYHA III–IV (vs II)	0.525	0.167	1.69 (1.22–2.34)	0.002
ln-NT-proBNP, per unit	0.315	0.072	1.37 (1.18–1.58)	<0.001
eGFR, per 10 mL/min/1.73 m²	−0.151	0.052	0.86 (0.78–0.95)	0.004
Uric acid, per 100 μmol/L	0.247	0.079	1.28 (1.10–1.49)	0.002
E/e′, per unit	0.039	0.017	1.04 (1.01–1.07)	0.018
LAVI, per 10 mL/m²	0.322	0.087	1.38 (1.16–1.64)	<0.001
HbA1c, per 1%	0.166	0.060	1.18 (1.05–1.33)	0.006

HR, hazard ratio; CI, confidence interval; SE, standard error. Abbreviations as in **Tables 1** and **2**.

### 3.6 Incremental predictive value of four-layer nested models

Results of the sequential four-layer nested Cox model comparison are presented in **Table 4**. Model 1 (age + NYHA + heart rate) yielded a C-index of 0.642 (95% CI 0.590–0.694). Addition of CKM-axis biomarkers (Model 2: + ln-NT-proBNP + uric acid) increased the C-index to 0.718 (ΔC = +0.076; continuous NRI = 0.448; IDI = 0.058; P < 0.001 by likelihood ratio test). Addition of diastolic echocardiographic parameters (Model 3: + E/e′ + LAVI) further increased the C-index to 0.784 (ΔC = +0.066; continuous NRI = 0.386; IDI = 0.072; P < 0.001). Finally, addition of HbA1c (Model 4: + HbA1c) raised the C-index to 0.812 (ΔC = +0.028; continuous NRI = 0.156; IDI = 0.026; P = 0.012), directly quantifying the incremental predictive value of HbA1c above the full CKM-axis and echocardiographic foundation.

**Table 4:** Incremental predictive value of four sequentially nested Cox models.

Model	Variables added	C-index (95% CI)	ΔC	NRI	IDI	LR-P	BIC
M1	Age + NYHA + HR	0.642 (0.590–0.694)	—	—	—	—	1162.0
M2	+ ln-NT-proBNP + UA	0.718 (0.668–0.768)	+0.076	0.448	0.058	<0.001	1125.9
M3	+ E/e′ + LAVI	0.784 (0.736–0.832)	+0.066	0.386	0.072	<0.001	1094.6
M4	+ HbA1c	0.812 (0.770–0.854)	+0.028	0.156	0.026	0.012	1091.9

ΔC, change in C-index versus prior model; NRI, continuous net reclassification improvement; IDI, integrated discrimination improvement; BIC, Bayesian information criterion; LR-P, likelihood ratio test P-value. HR, resting heart rate; UA, uric acid.

### 3.7 Model validation including temporal validation

**Figure 5:**
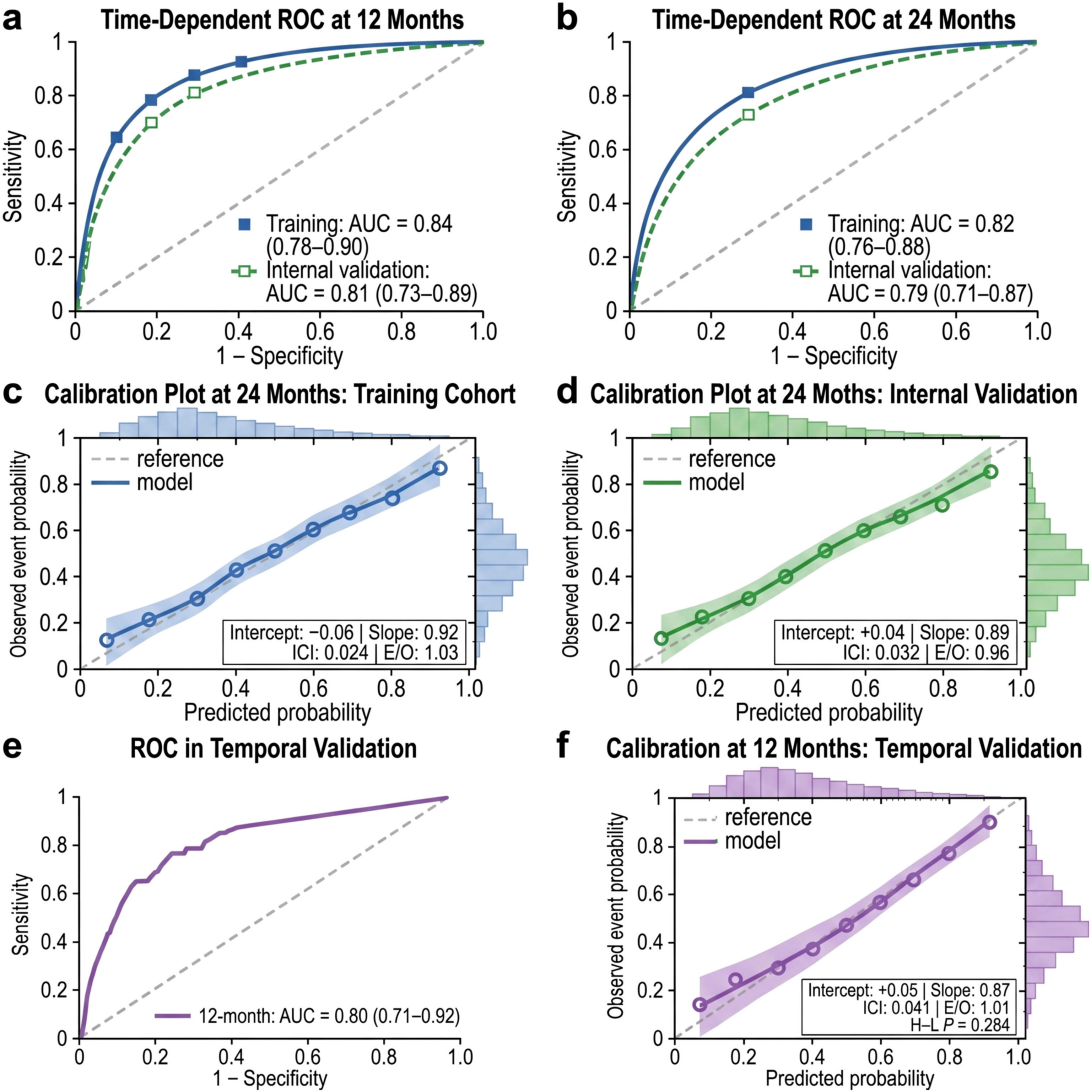
Model validation across development and temporal validation cohorts. **(a)** Time-dependent ROC curves at 12 months in the training (AUC 0.84) and internal validation (AUC 0.81) cohorts. **(b)** Time-dependent ROC curves at 24 months in the training (AUC 0.82) and internal validation (AUC 0.79) cohorts. **(c)** Calibration plot at 24 months in the training cohort (ICI = 0.024). **(d)** Calibration plot at 24 months in the internal validation cohort (ICI = 0.032). **(e)** ROC curves at 12 months in the temporal validation cohort (12-month AUC 0.80). **(f)** Calibration plot at 12 months in the temporal validation cohort (slope 0.87; ICI = 0.041).

In the development cohort, the nomogram achieved an apparent C-index of 0.812 (95% CI 0.770–0.854) in the training set and 0.790 (0.728–0.852) in the internal validation set. Time-dependent AUC values at 12 and 24 months were 0.84 (0.78–0.90) and 0.82 (0.76–0.88) in the training set, and 0.81 (0.73–0.89) and 0.79 (0.71–0.87) in the internal validation set, respectively (**Figures 5a, b**). Calibration metrics at 24 months demonstrated adequate performance: calibration-in-the-large intercept was −0.06 (training) and +0.04 (internal); calibration slope was 0.92 and 0.89; integrated calibration index (ICI) was 0.024 and 0.032; expected-to-observed ratio was 1.03 and 0.96 (**Figures 5c, d**). The 1,000-iteration bootstrap optimism-corrected C-index of the full modelling pipeline was 0.788 (optimism = 0.024), and the Brier score was 0.162 (training) and 0.174 (internal).

In the temporal validation cohort (n = 102; mean age 77.4 ± 7.1 years; 55.9% female; 38 endpoint events [37.3%]), baseline characteristics did not differ significantly from the development cohort except for a numerically higher SGLT2 inhibitor prescription rate (47.1% vs 39.1%; P = 0.162; **Supplementary Table 2**). In the temporal validation cohort, due to the chronological bounds of the study design (enrolment up to June 2025 and data lock on June 30, 2025), follow-up was capped at a maximum of 18 months. Consequently, to ensure statistical validity and prevent unwarranted model extrapolation, temporal validation was strictly restricted to the 12-month landmark. The model achieved a robust 12-month AUC of 0.80 in this temporal cohort. When the nomogram derived from the development cohort was applied without recalibration to the temporal cohort, discrimination was preserved: C-index 0.783 (0.718–0.848); 12-month AUC 0.80 (0.71–0.92) (**Figure 5e**). Calibration metrics showed mild attenuation consistent with expected performance degradation when applying a model to a later calendar period: calibration-in-the-large intercept +0.11; calibration slope 0.87; ICI 0.041; E/O ratio 0.92; Hosmer–Lemeshow P = 0.284 (**Figure 5f**; **Supplementary Figure 3**).

### 3.8 Clinical utility: decision curve analysis

**Figure 6:**
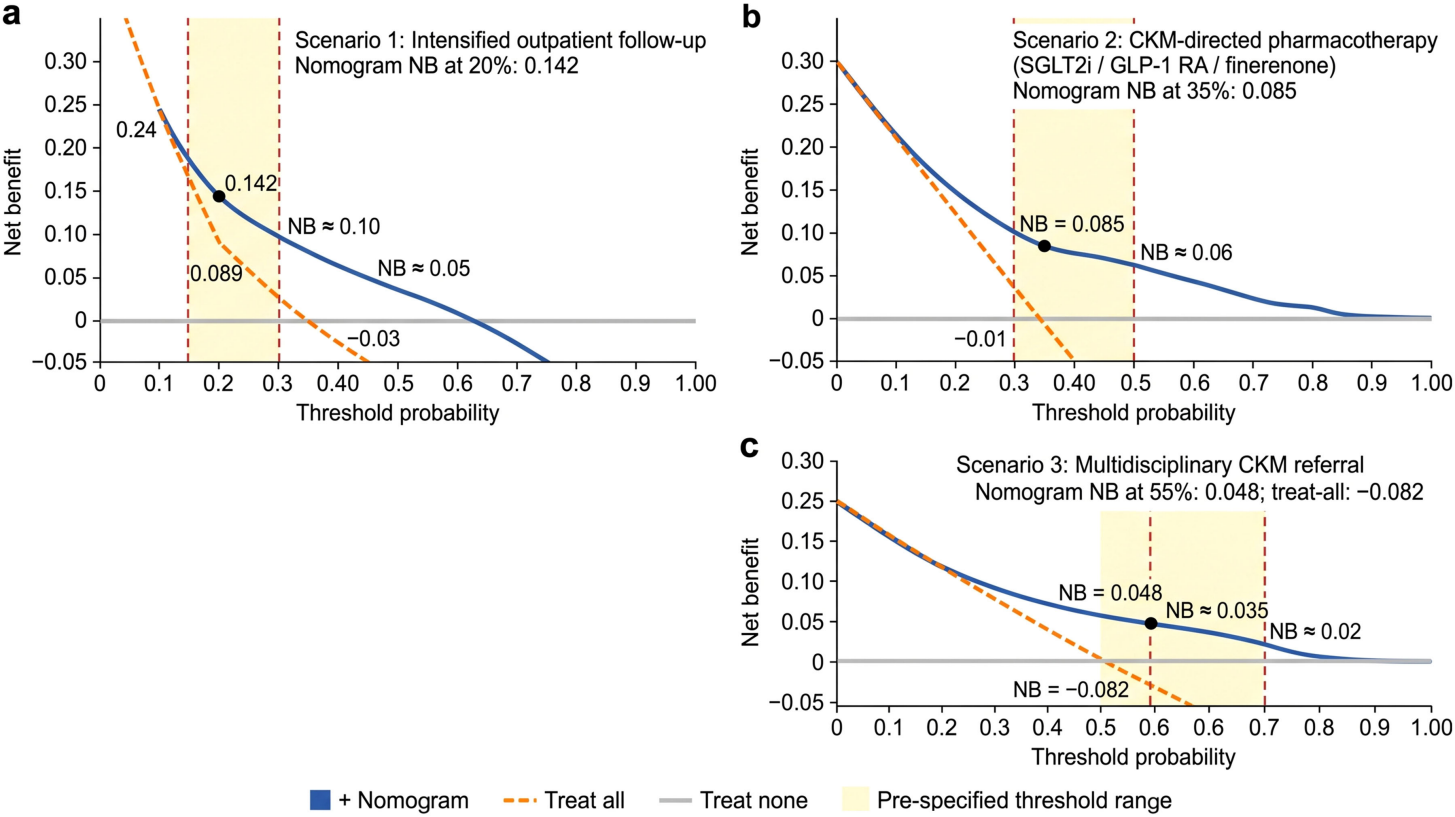
Decision curve analysis across three pre-specified clinical decision scenarios. **(a)** Scenario 1 — intensified outpatient follow-up (threshold probability 15–25%); mean net benefit of the nomogram 0.142 at 20% threshold versus 0.089 for treat-all and 0 for treat-none. **(b)** Scenario 2 — initiation of CKM-directed pharmacotherapy (threshold 30–45%); mean net benefit 0.085 at 35% threshold. **(c)** Scenario 3 — multidisciplinary CKM referral (threshold 50–65%); mean net benefit 0.048 at 55% threshold. Treat all” assumes all patients will experience the outcome, and “Treat none” assumes none will. The threshold range (10-85%) represents the clinically plausible spectrum of risk where intervention is acceptable.

Decision curve analyses across three pre-specified clinical decision scenarios are shown in **Figure 6**. In Scenario 1 (intensified outpatient follow-up; threshold probability 15–25%), the nomogram yielded a mean net benefit of 0.142 at the 20% threshold in the training cohort, compared with 0.089 for treat-all and 0 for treat-none (**Figure 6a**); net benefit at the same threshold in the internal validation cohort was 0.128. In Scenario 2 (initiation of CKM-directed pharmacotherapy including SGLT2 inhibitor or GLP-1 receptor agonist; threshold probability 30–45%), the nomogram yielded net benefit of 0.085 at the 35% threshold, and at thresholds above approximately 40% treat-all became net-harmful (**Figure 6b**). In Scenario 3 (multidisciplinary CKM referral; threshold probability 50–65%), the nomogram yielded net benefit of 0.048 at the 55% threshold whilst treat-all was markedly net-harmful (−0.082; **Figure 6c**). Across all three scenarios, the nomogram demonstrated positive net benefit exceeding both reference strategies.

To translate the nomogram’s predictive value into an actionable clinical threshold, we determined the optimal probability cut-off for the 24-month composite endpoint using the maximum Youden index. In the training cohort, the optimal predicted probability cut-off was 0.36. At this threshold, the nomogram demonstrated a sensitivity of 76.8%, a specificity of 74.4%, a positive predictive value (PPV) of 65.6%, and a negative predictive value (NPV) of 83.5%. Applying this identical threshold to the internal validation cohort yielded consistent diagnostic performance (sensitivity 73.5%, specificity 71.9%, PPV 61.0%, NPV 82.0%), confirming the model’s reliability for identifying patients at high risk of QoL deterioration, whilst the high NPV effectively rules out low-risk individuals. Importantly, to verify its incremental clinical value, our model was compared with the widely adopted MAGGIC risk score. The eight-variable CKM-based nomogram significantly outperformed the conventional MAGGIC score in predicting the 24-month composite endpoint (AUC 0.82 [95% CI 0.76–0.88] vs. MAGGIC AUC 0.63 [95% CI 0.57–0.69]; DeLong’s test P < 0.001). This comparison robustly underscores the necessity of incorporating CKM-axis biomarkers and glycaemic parameters (HbA1c) rather than relying solely on conventional clinical variables for risk stratification in this specific cardiometabolic HFpEF endotype.

### 3.9 CKM stage 4a metabolic burden stratification and GDMT balance

**Figure 7:**
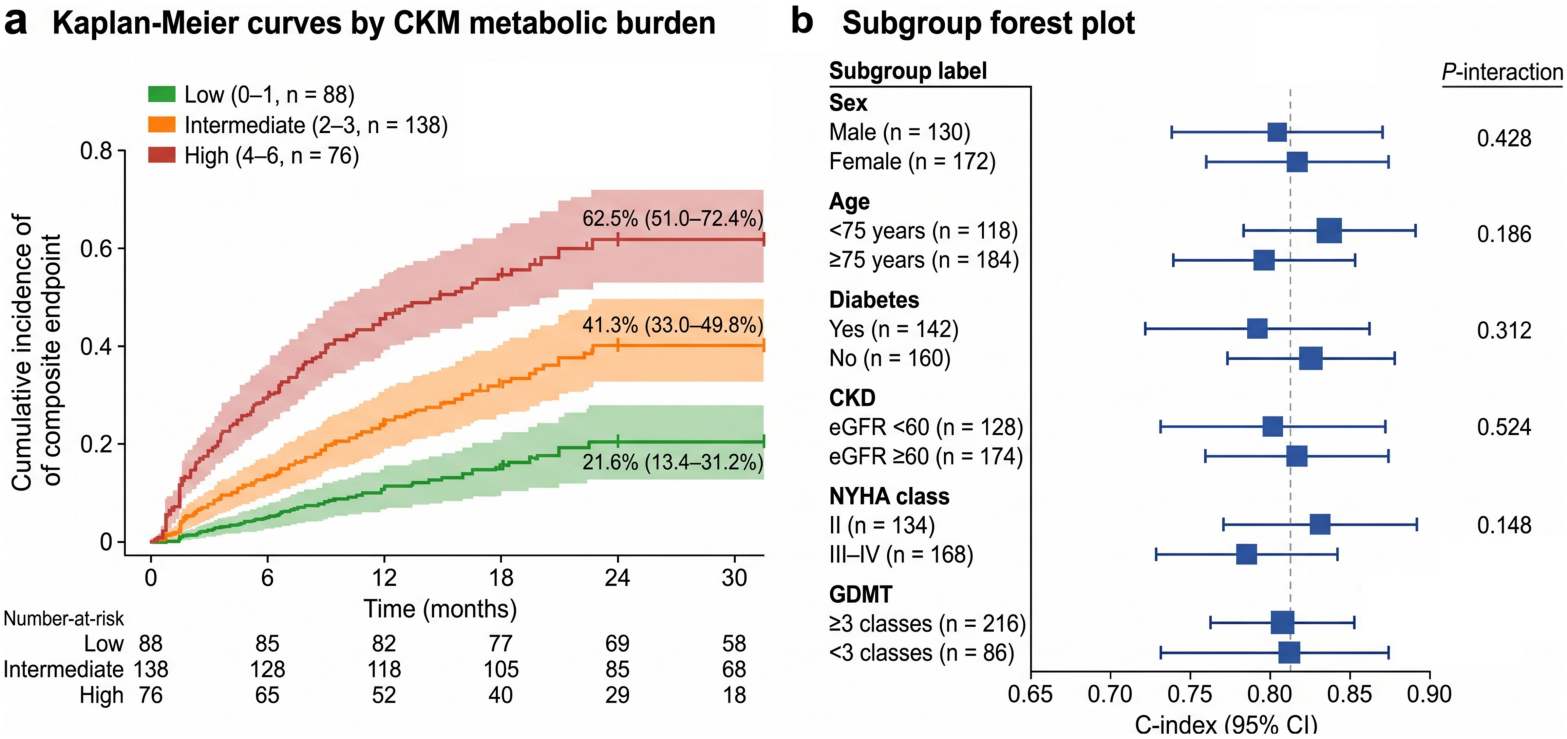
Within-stage stratification and subgroup performance. **(a)** Kaplan–Meier curves for the primary composite endpoint stratified by CKM stage 4a metabolic burden score (low 0–1, n = 88; intermediate 2–3, n = 138; high 4–6, n = 76). Log-rank χ² = 28.4; P < 0.001. 24-month cumulative incidences 21.6%, 41.3%, and 62.5%, respectively. **(b)** Forest plot of nomogram C-index across six pre-specified subgroups with interaction P-values. All P-interaction > 0.05.

Kaplan–Meier curves for the primary composite endpoint stratified by the CKM stage 4a metabolic burden score are shown in **Figure 7a**. The 24-month cumulative incidence was 21.6% (95% CI 13.4–31.2%) in the low-burden group (0–1 points; n = 88), 41.3% (33.0–49.8%) in the intermediate-burden group (2–3 points; n = 138), and 62.5% (51.0–72.4%) in the high-burden group (4–6 points; n = 76). Three-group separation was statistically significant (log-rank χ² = 28.4; P < 0.001). Mean HbA1c values across the three groups were 6.2%, 7.0%, and 8.2%, respectively, and mean TyG index values were 8.22, 8.64, and 9.18, respectively. Within-stratum nomogram C-indices were 0.78 (low), 0.80 (intermediate), and 0.76 (high). A Cox coefficient-weighted version of the score yielded a C-index of 0.813 versus 0.808 for the equal-weight score (ΔC = 0.005; P = 0.72), supporting the simpler equal-weight formulation.

Guideline-directed medical therapy prescription rates across burden strata are shown in **Table 5**. Rates of ACEI/ARB/ARNI, beta-blockers, mineralocorticoid receptor antagonists, SGLT2 inhibitors, finerenone, and loop diuretics did not differ significantly across burden strata (all P > 0.25). GLP-1 receptor agonists were prescribed preferentially in the high-burden stratum (15.8% vs 5.8% vs 4.5%; P = 0.009). The proportion of patients receiving at least three GDMT classes did not differ (70.5% vs 72.5% vs 71.1%; P = 0.937).

**Table 5:** Discharge guideline-directed medical therapy by CKM stage 4a metabolic burden stratum.

Medication class	Low burden (score 0–1; n = 88)	Intermediate burden (score 2–3; n = 138)	High burden (score 4–6; n = 76)	P value
Any ACEI, ARB, or ARNI	72 (81.8)	112 (81.2)	54 (71.1)	0.152
ACEI or ARB	56 (63.6)	86 (62.3)	42 (55.3)	0.491
ARNI (sacubitril-valsartan)	16 (18.2)	26 (18.8)	12 (15.8)	0.850
Beta-blocker	64 (72.7)	104 (75.4)	56 (73.7)	0.918
MRA (spironolactone or eplerenone)	48 (54.5)	78 (56.5)	44 (57.9)	0.898
SGLT2 inhibitor	34 (38.6)	56 (40.6)	28 (36.8)	0.856
GLP-1 receptor agonist	4 (4.5)	8 (5.8)	12 (15.8)	0.009
Finerenone	6 (6.8)	10 (7.2)	8 (10.5)	0.664
Loop diuretic	74 (84.1)	118 (85.5)	70 (92.1)	0.252
Thiazide or thiazide-like diuretic	10 (11.4)	20 (14.5)	12 (15.8)	0.716
Statin	62 (70.5)	92 (66.7)	52 (68.4)	0.836
Antiplatelet agent	38 (43.2)	58 (42.0)	30 (39.5)	0.879
Oral anticoagulant	28 (31.8)	40 (29.0)	26 (34.2)	0.716
≥3 guideline-directed classes	62 (70.5)	100 (72.5)	54 (71.1)	0.937
≥4 guideline-directed classes	38 (43.2)	60 (43.5)	32 (42.1)	0.977

Data are n (%). P values from Pearson χ² test or Fisher’s exact test (when expected cell count <5) comparing rates across the three CKM metabolic burden strata. Rates of all major guideline-directed medical therapy classes do not differ significantly across strata (all P ≥ 0.15) except for GLP-1 receptor agonists (P = 0.009), which were prescribed preferentially in the high-burden stratum, consistent with obesity and diabetes being explicit indications for this drug class. The overall proportion receiving ≥3 guideline-directed medical therapy classes did not differ across strata (70.5% vs 72.5% vs 71.1%; P = 0.937). Abbreviations as in **Table 1**.

### 3.10 Competing risk, subgroup and sensitivity analyses

The Fine–Gray subdistribution hazard model (**Supplementary Table 3**) yielded subdistribution hazard ratios that were directionally concordant with and numerically similar to cause-specific hazard ratios for all eight predictors, with a maximum absolute difference of 0.06 observed for ln-NT-proBNP. The 6-month landmark analysis excluding 18 patients with early events yielded a landmark C-index of 0.80 (95% CI 0.74–0.86). Forest plot subgroup analyses (**Figure 7b**) showed preserved nomogram C-index across six pre-specified strata: male 0.806 vs female 0.818 (P-interaction = 0.428); age <75 years 0.824 vs ≥75 years 0.798 (P = 0.186); diabetic 0.794 vs non-diabetic 0.828 (P = 0.312); eGFR <60 0.802 vs ≥60 0.820 (P = 0.524); NYHA II 0.836 vs III–IV 0.790 (P = 0.148); and GDMT ≥3 classes 0.808 vs <3 classes 0.814 (P = 0.682). No statistically significant interaction was detected. The MLHFQ threshold sensitivity analysis (**Table 6**) demonstrated preserved discrimination (training C-index 0.798–0.826; internal-validation C-index 0.774–0.804) and calibration (calibration slope 0.90–0.94) across all four alternative endpoint definitions. The pooled hazard ratios and P values for the eight LASSO-selected predictors under each endpoint definition are provided in **Supplementary Table 4**. The direction and magnitude of all eight hazard ratios were stable across definitions. Seven of the eight predictors retained statistical significance (P < 0.05) under all four definitions; HbA1c remained significant under three definitions (≥3 points, ≥5 points, and ≥10% relative change) but attenuated to borderline non-significance under the more specific ≥7-point threshold (HR 1.14, 95% CI 0.99–1.30, P = 0.060), consistent with the smaller number of events (n = 92) and correspondingly reduced statistical power under the most stringent definition.

**Table 6:** MLHFQ threshold sensitivity analysis across four alternative endpoint operationalisations.

MLHFQ endpoint definition	Events (%)	Training C-index (95% CI)	Internal C-index (95% CI)	Calibration slope
≥3 points (more sensitive)	138 (45.7)	0.798 (0.754–0.842)	0.774 (0.708–0.840)	0.94
≥5 points (primary)	116 (38.4)	0.812 (0.770–0.854)	0.790 (0.728–0.852)	0.92
≥7 points (more specific)	92 (30.5)	0.826 (0.784–0.868)	0.804 (0.740–0.868)	0.90
≥10% relative change	108 (35.8)	0.803 (0.759–0.847)	0.786 (0.722–0.850)	0.93

Event rates, discrimination, and calibration were preserved across all four definitions, and all eight predictors retained statistical significance showed in **Table 3**.

## 4 Discussion

In this retrospective cohort of elderly CKM stage 4a HFpEF patients, we developed and internally validated a LASSO-Cox nomogram incorporating eight routinely obtainable baseline variables — age, NYHA class, ln-NT-proBNP, eGFR, uric acid, E/e′ ratio, LAVI, and HbA1c — that predicted 12- and 24-month quality-of-life deterioration with a C-index of 0.812 (95% CI 0.770–0.854) in the training set and 0.790 (0.728–0.852) in the internal validation set. In a separate temporal validation cohort of 102 consecutive patients admitted during a later non-overlapping period, the nomogram retained a C-index of 0.783 (0.718–0.848) without recalibration, with an integrated calibration index (ICI) of 0.041 and preserved net benefit across three pre-specified clinical decision scenarios. HbA1c was the only glycaemic metabolism indicator retained by LASSO; LVEF was uninformative; and the four-layer nested model showed that HbA1c yielded a statistically significant incremental continuous net reclassification improvement of 0.156 (P = 0.012) above the combined CKM-axis and echocardiographic foundation. An exploratory CKM stage 4a metabolic burden score stratified patients into subgroups with 24-month event rates of 21.6%, 41.3%, and 62.5%.

Each selected predictor maps to one or more axes of CKM syndrome as defined by the 2023 American Heart Association Presidential Advisory (4). Ln-NT-proBNP is the canonical cardiovascular-axis biomarker, reflecting myocardial wall stress and neurohormonal activation. E/e′ and LAVI constitute the echocardiographic embodiment of the cardiovascular axis in HFpEF: E/e′ captures instantaneous left ventricular filling pressure, whereas LAVI integrates the chronic haemodynamic burden of elevated filling pressure over time. Our observed independent adjusted hazard ratios of 1.04 per unit for E/e′ and 1.38 per 10 mL/m² for LAVI are consistent with the prior PROMIS-HFpEF analysis (n = 1,154), which identified these parameters as the strongest imaging-derived predictors of adverse outcomes in HFpEF (21). eGFR and uric acid jointly represent the renal axis, with uric acid occupying a dual-axis position as a product of purine catabolism and an active substrate for xanthine oxidase-mediated reactive oxygen species generation (22). HbA1c represents the metabolic axis and constitutes the principal mechanistic contribution of this model. Age captures cumulative lifetime CKM exposure, and NYHA class serves as a functional integrator of multisystem burden. Collectively, these eight variables span all three CKM axes and cover four distinct information domains: clinical assessment, circulating biomarkers, echocardiographic imaging, and glycaemic metabolism.

The selection of HbA1c by LASSO — with simultaneous exclusion of fasting glucose, TyG index, and HGI — is the most clinically consequential finding of this study. HbA1c is a time-integrated metric of glycaemic exposure over the preceding 2–3 months, and its predictive superiority over point-in-time indicators (fasting glucose) and derived indices (TyG, HGI) suggests that cumulative chronic hyperglycaemic burden is a stronger determinant of quality-of-life trajectory in HFpEF than instantaneous insulin resistance or non-glycaemic glycation. After adjustment for all other LASSO-selected variables, HbA1c retained moderate-to-strong partial correlations with TyG (r = 0.58) and HGI (r = 0.63), confirming that HbA1c subsumes the information content of both derived indices.

From a mechanistic perspective, HbA1c mediates diastolic dysfunction in cardiometabolic HFpEF through at least three molecular pathways active in this endotype (23–25). First, advanced glycation end-product (AGE) accumulation promotes irreversible cross-linking of type I and III collagen fibrils in the myocardial interstitium, increasing passive chamber stiffness. Second, AGE accumulation and concurrent systemic insulin resistance impair nitric oxide–cyclic guanosine monophosphate–protein kinase G (NO–cGMP–PKG) signalling in cardiomyocytes; van Heerebeek and colleagues demonstrated that lower myocardial PKG activity in HFpEF associates with higher cardiomyocyte resting tension and increased nitrosative stress (26). PKG-dependent hypophosphorylation of the titin N2Bus unique-sequence domain is now recognised as the predominant titin-based mechanism of increased cardiomyocyte passive stiffness in HFpEF, with a concurrent shift in titin isoform composition from the more compliant N2BA towards the stiffer N2B variant as a secondary contributor (27, 28). Third, chronic hyperglycaemia drives mitochondrial substrate utilisation towards fatty acid beta-oxidation and generates lipotoxic intermediates that impair myocardial metabolic flexibility, a hallmark of the cardiometabolic HFpEF endotype (25). These three mechanisms converge to render HbA1c a particularly potent predictor in HFpEF, where diastolic stiffness rather than systolic pump failure constitutes the primary haemodynamic lesion. Our findings extend the CHARM observation that HbA1c exhibits a graded relationship with cardiovascular death or heart failure hospitalisation, which was paradoxically more pronounced in non-diabetic than in diabetic patients (29).

The absorption of HGI by HbA1c also deserves careful biological interpretation. HGI — originally characterised by Hempe and colleagues as the portion of observed HbA1c not explained by ambient plasma glucose (30) — reflects several interacting biological determinants, including inter-individual variation in erythrocyte lifespan (31), differences in intra-erythrocyte glucose metabolism, and systemic oxidative stress. In the continuous glucose monitoring era, the concept has been refined into the glycation gap and glucose management indicator (GMI) framework (32). Our observation that HGI is absorbed by HbA1c under LASSO does not imply that HGI is mechanistically redundant; rather, HbA1c already integrates the summed glycation exposure from these pathways, so adding HGI as a residual of HbA1c contributes limited incremental information when HbA1c is retained.

A defining feature of our LASSO results is the complete compression of LVEF to a zero coefficient across all five imputed datasets. This finding is an inherent consequence of the study design: in a pure HFpEF cohort (LVEF ≥50% by definition), inter-patient variance in LVEF is markedly compressed (typically 50–75%), leaving negligible discriminatory information. This observation aligns with the broader recognition that LVEF-based classification fails to capture the pathophysiological complexity of HFpEF (33). The concurrent retention of E/e′ and LAVI by LASSO supports a shift from systolic-function-centric metrics to diastolic-function-driven phenotyping, consistent with the 2023 ACC Expert Consensus Decision Pathway, which recommended E/e′ and LAVI as the primary echocardiographic targets for HFpEF evaluation (12). Our adjusted hazard ratios of 1.04 per unit increase in E/e′ and 1.38 per 10 mL/m² increase in LAVI translate the paradigm’s mechanistic content into quantified prognostic weights.

The U-shaped restricted cubic spline relationship between uric acid and quality-of-life deterioration (P-nonlinear = 0.006; nadir near 350 μmol/L) deserves specific comment. The ascending right limb (uric acid >450 μmol/L) reflects the established pro-oxidant and pro-inflammatory effects of hyperuricaemia, mediated by xanthine oxidase activation, endothelial dysfunction, and renal afferent arteriolar vasoconstriction (22, 34). The ascending left limb (uric acid <280 μmol/L) likely reflects the reverse epidemiology pattern previously reported in chronic heart failure, whereby low uric acid accompanies poor nutritional status, sarcopenia, and hepatic synthetic failure (35). This biphasic pattern, previously reported in the URRAH cohort (n = 22,714) and in multiple Asian heart failure registries (34), supports the interpretation that uric acid functions as a bidirectional biomarker within the CKM framework: its prognostic interpretation shifts from metabolic excess to metabolic depletion depending on the patient’s position on the CKM severity spectrum. The observed nadir at approximately 350 μmol/L in the present study provides a candidate reference value for future prospective validation.

The choice of quality-of-life deterioration as the primary composite endpoint deserves specific justification because most HFpEF prediction models have targeted hard endpoints such as cardiovascular mortality or heart failure hospitalisation. Three considerations support our design choice in the CKM stage 4a elderly population. First, quality of life has been adopted as a co-primary therapeutic target in current HFpEF guidelines (12) and in two of the three major HFpEF therapeutic programmes of the past five years: the STEP-HFpEF and STEP-HFpEF DM trials used Kansas City Cardiomyopathy Questionnaire Clinical Summary Score (KCCQ-CSS) as a primary endpoint (13, 36), and FINEARTS-HF included KCCQ in the secondary endpoint hierarchy (14, 37). Second, in CKM stage 4a HFpEF, quality of life is mechanistically downstream of all three CKM axes simultaneously: glycaemic dysregulation drives fatigue and neuropathic symptoms, renal impairment contributes to volume overload and lethargy, and diastolic dysfunction produces the cardinal symptoms of dyspnoea and exercise intolerance. A patient-reported outcome therefore functions as an integrated downstream readout of CKM axis dysfunction. Third, in an elderly population where absolute survival gains from any single intervention are modest, improvements or deteriorations in quality of life carry greater marginal meaning to individual patients than changes in hard clinical events. We therefore position the present nomogram as complementary to, rather than competing with, existing mortality-focused HFpEF prediction tools.

The SGLT2 inhibitor interaction analysis revealed a numerically lower 24-month event rate in SGLT2 inhibitor users compared with non-users (30.5% vs 42.9%). However, the formal interaction term did not reach statistical significance (P-interaction = 0.14), and we interpret this observation as hypothesis-generating only, for three reasons: (i) SGLT2 inhibitor prescription was not randomised and is subject to confounding by indication; (ii) the sample size is insufficient to reliably detect a treatment-by-risk interaction of plausible magnitude; and (iii) discharge prescription does not capture adherence or dose changes during follow-up. The mechanistic plausibility of SGLT2 inhibitor benefit in CKM stage 4a HFpEF is nonetheless well established through randomised evidence. EMPEROR-Preserved and DELIVER demonstrated that empagliflozin and dapagliflozin reduced the composite of cardiovascular death or heart failure hospitalisation in HFpEF populations (38, 39). Mechanistically, SGLT2 inhibitors simultaneously attenuate tubuloglomerular feedback, reduce epicardial adipose tissue volume and systemic inflammation, and decrease left ventricular filling pressures through osmotic natriuresis (40).

The CKM metabolic burden score identifies patients in the high-burden stratum (score 4–6; 24-month event rate 62.5%) who might derive the greatest absolute benefit from multi-organ protective strategies. GLP-1 receptor agonists, supported by the pooled STEP-HFpEF and STEP-HFpEF DM analyses in obesity-related HFpEF (36), were already prescribed preferentially in the high-burden stratum in our cohort (15.8% vs 5.8% vs 4.5%; P = 0.009), suggesting that real-world clinical allocation is already tracking the CKM burden gradient to some extent. Finerenone, which demonstrated HFpEF-specific efficacy in the FINEARTS-HF trial, offers a third CKM-directed option whose benefit was preserved across the spectrum of PREDICT-HFpEF-estimated baseline risk in a recent secondary analysis (14, 41). The nomogram risk score could plausibly serve as an enrichment tool for future randomised trials evaluating whether high-risk CKM stage 4a HFpEF patients derive disproportionate benefit from SGLT2 inhibitors, GLP-1 receptor agonists, or finerenone.

The degraded performance of the MAGGIC score (AUC = 0.63) in our cohort highlights a critical pathophysiological distinction: because the MAGGIC score was derived primarily from general HF populations (predominantly HFrEF), it lacks essential echocardiographic diastolic parameters (e.g., E/e′, LAVI) and cumulative glycaemic indicators (HbA1c), which are the core drivers of cardiometabolic HFpEF progression. Furthermore, it is crucial to contextualise the specific clinical setting of our predictive model. While acute severity scores such as SOFA and SAPS II are the gold standards for predicting short-term hard endpoints (e.g., in-hospital or 28-day mortality) in critically ill intensive care unit (ICU) patients, they are methodologically misaligned with the stable, ambulatory HFpEF population. In the CKM stage 4a trajectory, the primary clinical burden manifests as chronic metabolic remodelling and insidious functional decline rather than acute multi-organ failure. Because stable HFpEF patients typically lack severe acute physiological derangements, applying ICU-centric scores would inevitably result in significant floor effects and poor discrimination. By directly outperforming traditional outpatient HF scores in our specific cohort, our nomogram demonstrates profound incremental value tailored precisely to the chronic cardiometabolic HFpEF phenotype.

Our nomogram’s performance (training C-index 0.812; temporal validation 0.783) is broadly consistent with published HFpEF prediction tools whilst addressing three gaps. The PREDICT-HFpEF model, derived from the DELIVER trial (n = 6,263) and validated across PARAGON-HF and I-PRESERVE, achieved 1-year C-statistics of 0.71–0.75 for the composite of heart failure hospitalisation or cardiovascular death; its 11 variables include diabetes status as a binary term but do not include HbA1c or echocardiographic diastolic function parameters (8). A recent secondary analysis of PREDICT-HFpEF in the FINEARTS-HF trial reported preserved discrimination and demonstrated that the benefit of finerenone was consistent across baseline risk quartiles (41). Other recent models based on electronic health records have reported 1-year mortality areas under the curve of 0.78–0.83 in HFpEF, with age and NT-proBNP consistently identified as the top-ranking features (11, 42). The Zhou et al. LASSO nomogram for HFpEF rehospitalisation (n = 1,149) included the TyG index and E/e′ but omitted HbA1c and LAVI (9). Our model differs in three respects: (i) it incorporates HbA1c as a continuous predictor alongside E/e′ and LAVI within a CKM-anchored architecture; (ii) it is explicitly positioned within the AHA CKM stage 4a framework; and (iii) it uses quality-of-life deterioration as the primary endpoint, a patient-centred target increasingly prioritised in HFpEF therapeutic development.

### 4.1 Strengths and limitations

This study has several strengths. First, restriction to a pure HFpEF cohort (LVEF ≥50%) eliminates phenotypic heterogeneity that confounds studies of mixed HF populations. Second, LASSO-driven variable selection integrated with multiple imputation avoids the investigator bias inherent in stepwise regression. Third, the explicit CKM stage 4a framing provides a pathophysiological rationale for predictor selection. Fourth, the four-layer nested model analysis transparently decomposes the incremental value of each information domain. Fifth, the inclusion of a temporal validation cohort (n = 102) is a methodological advance over pure internal validation; the nomogram retained a C-index of 0.783 with preserved calibration (ICI 0.041) and net benefit across three clinical decision scenarios when applied without recalibration. Sixth, the comprehensive validation framework aligns with the TRIPOD+AI 2024 reporting standard (16) and addresses the four Prediction model Risk Of Bias ASsessment Tool (PROBAST) domains (43); bootstrap optimism correction of the full modelling pipeline (optimism = 0.024) mitigates overfitting concerns, and calibration reporting followed the Van Calster hierarchy (44).

Several limitations should be acknowledged. First, the development cohort is single-centre and retrospective, and the temporal validation cohort — whilst enrolled under identical criteria during a non-overlapping later period — was recruited from the same institution, sharing practice patterns, referral pathways, and patient demographics. Prospective multicentre geographic external validation remains essential before clinical deployment. Second, the moderate sample size, whilst adequate for an 8-variable model based on Riley criteria (18), may have been insufficient to detect statistically significant SGLT2 inhibitor interactions of plausible magnitude. Third, urinary albumin-to-creatinine ratio — a key CKM biomarker in the AHA advisory — was not available in our dataset, potentially underrepresenting the renal axis; our study consequently used eGFR + clinical cardiovascular disease + metabolic risk factors as an operationalised definition of CKM stage 4a. Fourth, the CKM metabolic burden score is an exploratory descriptive tool; whilst sensitivity analyses confirmed threshold robustness and Cox-coefficient weighting offered no performance advantage (ΔC = 0.005; P = 0.72), external validation is a prerequisite for clinical adoption. Calibration slopes marginally below 1.0 (0.89–0.92 across validation cohorts) suggest mild overfitting inherent to any prediction model developed in a single-centre cohort of moderate size; shrinkage-adjusted recalibration would be appropriate before clinical deployment. Fifth, MLHFQ assessments were performed approximately every 12 months, which may miss quality-of-life fluctuations occurring between assessment windows. Sixth, baseline laboratory values and echocardiographic parameters obtained at a single admission timepoint may not fully represent longitudinal disease trajectory. Seventh, it is important to explicitly define the clinical boundaries of our model’s generalizability. This nomogram was specifically developed and validated for stable or ambulatory elderly CKM stage 4a HFpEF patients, deliberately focusing on long-term patient-centred outcomes (QoL deterioration and chronic rehospitalisation) (45). Consequently, owing to the non-ICU nature of our cohort (which, by AHA definition, explicitly excludes patients with kidney failure requiring dialysis), ultra-short-term endpoints (such as in-hospital or 28-day mortality) and acute critical care interventions (such as continuous renal replacement therapy and invasive mechanical ventilation) were exceedingly rare. Predicting these acute endpoints was not statistically feasible due to severe event-per-variable constraints. Our nomogram is exclusively designed for long-term outpatient risk stratification and should not be generalised to acute cardiogenic shock management or ICU prognostication. Finally, our findings regarding the attenuation of risk in patients receiving SGLT2 inhibitors or GLP-1 receptor agonists are strictly observational and hypothesis-generating. Due to the inherent confounding by indication in retrospective designs, this analysis does not prove causal treatment efficacy and should not be interpreted as substitute evidence for randomised controlled trials.

## 5 Conclusions

In elderly CKM stage 4a HFpEF patients, an eight-variable LASSO-Cox nomogram integrating CKM-axis biomarkers, glycaemic metabolism (HbA1c), and echocardiographic diastolic function parameters (E/e′ and LAVI) predicted long-term composite endpoint (quality-of-life deterioration or unplanned heart failure rehospitalisation) with preserved performance in temporal validation. HbA1c emerged as a novel independent predictor that subsumed the information carried by the TyG index and HGI. LVEF provided no prognostic information in this pure HFpEF cohort, reinforcing the need for a diastolic-phenotype-driven approach to risk assessment. The exploratory CKM metabolic burden score demonstrated the feasibility of within-stage risk stratification and may guide enrichment for future trials of CKM-directed therapies. Prospective multicentre geographic external validation is warranted.

## Data Availability

The original contributions presented in the study are included in the article/**Supplementary Material**. Further inquiries can be directed to the corresponding author.

## Supplementary material

Supplementary Figure 1 Kernel density overlay plots comparing observed and imputed distributions for six variables with missing rates >5%.

Supplementary Figure 2 LASSO variable selection frequency heatmap across five imputed datasets at λ.min.

Supplementary Figure 3 Temporal validation calibration plot at 18 months with 1,000-bootstrap resampling.

Supplementary Table 1 Variable-specific missing data rates and MICE quality indicators.

Supplementary Table 2 Baseline characteristics comparing development cohort with temporal validation cohort.

Supplementary Table 3 Fine–Gray subdistribution hazard model results.

Supplementary Table 4 Pooled hazard ratios for the eight LASSO-selected predictors under four MLHFQ endpoint definitions.

## References

[1] Savarese G Becher PM Lund LH Seferovic P Rosano GMC Coats AJS . Global burden of heart failure: a comprehensive and updated review of epidemiology. Cardiovasc Res. (2023) 118:3272–87. doi: 10.1093/cvr/cvac013 35150240

[2] Martin SS Aday AW Almarzooq ZI Anderson CAM Arora P Avery CL . 2024 heart disease and stroke statistics: a report of US and global data from the American Heart Association. Circulation. (2024) 149:e347–913. doi: 10.1161/CIR.0000000000001209 38264914 PMC12146881

[3] Borlaug BA Sharma K Shah SJ Ho JE . Heart failure with preserved ejection fraction: JACC scientific statement. J Am Coll Cardiol. (2023) 81:1810–34. doi: 10.1016/j.jacc.2023.01.049 37137592

[4] Ndumele CE Rangaswami J Chow SL Neeland IJ Tuttle KR Khan SS . Cardiovascular-kidney-metabolic health: a presidential advisory from the American Heart Association. Circulation. (2023) 148:1606–35. doi: 10.1161/CIR.0000000000001184 37807924

[5] Packer M . What exactly is cardiometabolic HFpEF: a phenotype or an endotype? Circ Heart Fail. (2026) 19:e014031. doi: 10.1161/CIRCHEARTFAILURE.125.014031 41574415 PMC12986044

[6] Gorica E Geiger MA Di Venanzio L Atzemian N Kleeberger JA Grigorian D . Cardiometabolic heart failure with preserved ejection fraction: from molecular signatures to personalized treatment. Cardiovasc Diabetol. (2025) 24:265. doi: 10.1186/s12933-025-02774-w 40611109 PMC12225509

[7] Verma R Dhingra NK Connelly KA . Obesity/cardiometabolic phenotype of heart failure with preserved ejection fraction: mechanisms to recent trials. Curr Opin Cardiol. (2024) 39:92–7. doi: 10.1097/HCO.0000000000001113 38294186

[8] McDowell K Kondo T Talebi A Teh K Bachus E de Boer RA . Prognostic models for mortality and morbidity in heart failure with preserved ejection fraction. JAMA Cardiol. (2024) 9:457–65. doi: 10.1001/jamacardio.2024.0284 38536153 PMC10974691

[9] Zhou Y Wang H Chen J Li Q Liu Y Zhang L . A web-based dynamic nomogram for predicting readmission in patients with heart failure with preserved ejection fraction. Front Cardiovasc Med. (2025) 12:1492717. doi: 10.3389/fcvm.2025.1492717 40606022 PMC12213557

[10] Pocock SJ Ariti CA McMurray JJV Maggioni A Køber L Squire IB . Predicting survival in heart failure: a risk score based on 39 372 patients from 30 studies. Eur Heart J. (2013) 34:1404–13. doi: 10.1093/eurheartj/ehs337 23095984

[11] Shin I Bhatt N Alashi A Kandala K Murugiah K . Predicting 30-day and 1-year mortality in heart failure with preserved ejection fraction (HFpEF). PloS One. (2025) 20:e0336809. doi: 10.1371/journal.pone.0336809 41237168 PMC12617840

[12] Kittleson MM Panjrath GS Amancherla K Davis LL Deswal A Dixon DL . 2023 ACC Expert Consensus Decision Pathway on management of heart failure with preserved ejection fraction: a report of the American College of Cardiology Solution Set Oversight Committee. J Am Coll Cardiol. (2023) 81:1835–78. doi: 10.1016/j.jacc.2023.03.393 37137593

[13] Kosiborod MN Abildstrøm SZ Borlaug BA Butler J Rasmussen S Davies M . Semaglutide in patients with heart failure with preserved ejection fraction and obesity. N Engl J Med. (2023) 389:1069–84. doi: 10.1056/NEJMoa2306963 37622681

[14] Solomon SD McMurray JJV Vaduganathan M Claggett B Jhund PS Desai AS . Finerenone in heart failure with mildly reduced or preserved ejection fraction. N Engl J Med. (2024) 391:1475–85. doi: 10.1056/NEJMoa2407107 39225278

[15] Ju L Ji R Meng H Li P Du J Wang Y . Spatiotemporal deep video-phenomapping decodes microvascular rarefaction in middle-aged and elder renovascular hypertension: a multi-modal study integrating spatial transcriptomics and mitochondrial pyroptosis. Research (Wash D C). (2026) 9:1339. doi: 10.34133/research.1339 42404353 PMC13329644

[16] Collins GS Moons KGM Dhiman P Riley RD Beam AL Van Calster B . TRIPOD+AI statement: updated guidance for reporting clinical prediction models that use regression or machine learning methods. BMJ. (2024) 385:e078378. doi: 10.1136/bmj-2023-078378 38626948 PMC11019967

[17] McDonagh TA Metra M Adamo M Gardner RS Baumbach A Böhm M . 2021 ESC Guidelines for the diagnosis and treatment of acute and chronic heart failure. Eur Heart J. (2021) 42:3599–726. doi: 10.1093/eurheartj/ehab368 34922348

[18] Riley RD Ensor J Snell KIE Harrell FE Jr Martin GP Reitsma JB . Calculating the sample size required for developing a clinical prediction model. BMJ. (2020) 368:m441. doi: 10.1136/bmj.m441 32188600

[19] Inker LA Eneanya ND Coresh J Tighiouart H Wang D Sang Y . New creatinine- and cystatin C-based equations to estimate GFR without race. N Engl J Med. (2021) 385:1737–49. doi: 10.1056/NEJMoa2102953 34554658 PMC8822996

[20] Lang RM Badano LP Mor-Avi V Afilalo J Armstrong A Ernande L . Recommendations for cardiac chamber quantification by echocardiography in adults. J Am Soc Echocardiogr. (2015) 28:1–39.e14. doi: 10.1016/j.echo.2014.10.003 25559473

[21] Shah SJ Lam CSP Svedlund S Saraste A Hage C Tan RS . Prevalence and correlates of coronary microvascular dysfunction in heart failure with preserved ejection fraction: PROMIS-HFpEF. Eur Heart J. (2018) 39:3439–50. doi: 10.1093/eurheartj/ehy531 30165580 PMC6927847

[22] Hare JM Johnson RJ . Uric acid predicts clinical outcomes in heart failure: insights regarding the role of xanthine oxidase and uric acid in disease pathophysiology. Circulation. (2003) 107:1951–3. doi: 10.1161/01.CIR.0000066420.36123.35 12707249

[23] Paulus WJ Tschöpe C . A novel paradigm for heart failure with preserved ejection fraction: comorbidities drive myocardial dysfunction and remodeling through coronary microvascular endothelial inflammation. J Am Coll Cardiol. (2013) 62:263–71. doi: 10.1016/j.jacc.2013.02.092 23684677

[24] Schiattarella GG Altamirano F Tong D French KM Villalobos E Kim SY . Nitrosative stress drives heart failure with preserved ejection fraction. Nature. (2019) 568:351–6. doi: 10.1038/s41586-019-1100-z 30971818 PMC6635957

[25] ZhangH SunL XuT HouX ZhongF ShiF . ACADM-mediated fatty acid β-oxidation pathway in atherosclerosis and abdominal aortic aneurysm. Eur Heart J. (2026) ehag372. doi: 10.1093/eurheartj/ehag372 42247143

[26] van Heerebeek L Hamdani N Falcão-Pires I Leite-Moreira AF Begieneman MP Bronzwaer JG . Low myocardial protein kinase G activity in heart failure with preserved ejection fraction. Circulation. (2012) 126:830–9. doi: 10.1161/CIRCULATIONAHA.111.076075 22806632

[27] Borbély A Falcão-Pires I van Heerebeek L Hamdani N Édes I Gavina C . Hypophosphorylation of the stiff N2B titin isoform raises cardiomyocyte resting tension in failing human myocardium. Circ Res. (2009) 104:780–6. doi: 10.1161/CIRCRESAHA.108.193326 19179657

[28] Hamdani N Franssen C Lourenço A Falcão-Pires I Fontoura D Leite S . Myocardial titin hypophosphorylation importantly contributes to heart failure with preserved ejection fraction in a rat metabolic risk model. Circ Heart Fail. (2013) 6:1239–49. doi: 10.1161/CIRCHEARTFAILURE.113.000539 24014826

[29] Gerstein HC Swedberg K Carlsson J McMurray JJV Michelson EL Olofsson B . The hemoglobin A1c level as a progressive risk factor for cardiovascular death, hospitalization for heart failure, or death in patients with chronic heart failure: an analysis of the CHARM program. Arch Intern Med. (2008) 168:1699–704. doi: 10.1001/archinte.168.15.1699 18695086

[30] Hempe JM Gomez R McCarter RJ Jr Chalew SA . High and low hemoglobin glycation phenotypes in type 1 diabetes. J Diabetes Complications. (2002) 16:313–20. doi: 10.1016/s1056-8727(01)00227-6 12200073

[31] Cohen RM Franco RS Khera PK Smith EP Lindsell CJ Ciraolo PJ . Red cell life span heterogeneity in hematologically normal people is sufficient to alter HbA1c. Blood. (2008) 112:4284–91. doi: 10.1182/blood-2008-04-154112 18694998 PMC2581997

[32] Bergenstal RM Beck RW Close KL Grunberger G Sacks DB Kowalski A . Glucose management indicator (GMI): a new term for estimating A1C from continuous glucose monitoring. Diabetes Care. (2018) 41(11):2275–80. doi: 10.2337/dc18-1581 30224348 PMC6196826

[33] Borlaug BA . Evaluation and management of heart failure with preserved ejection fraction. Nat Rev Cardiol. (2020) 17:559–73. doi: 10.1038/s41569-020-0363-2 32231333

[34] Virdis A Masi S Casiglia E Tikhonoff V Cicero AFG Ungar A . Identification of the uric acid thresholds predicting an increased total and cardiovascular mortality over 20 years. Hypertension. (2020) 75:302–8. doi: 10.1161/HYPERTENSIONAHA.119.13643 31813345

[35] Anker SD Doehner W Rauchhaus M Sharma R Francis D Knosalla C . Uric acid and survival in chronic heart failure: validation and application in metabolic, functional, and hemodynamic staging. Circulation. (2003) 107:1991–7. doi: 10.1161/01.CIR.0000065637.10517.A0 12707250

[36] Butler J Shah SJ Petrie MC Borlaug BA Abildstrøm SZ Davies MJ . Semaglutide versus placebo in people with obesity-related heart failure with preserved ejection fraction: a pooled analysis of the STEP-HFpEF and STEP-HFpEF DM randomised trials. Lancet. (2024) 403:1635–48. doi: 10.1016/S0140-6736(24)00469-0 38599221 PMC11317105

[37] Desai AS Vaduganathan M Claggett BL Kulac IJ Jhund PS Cunningham J . Finerenone in patients with a recent worsening heart failure event: the FINEARTS-HF trial. J Am Coll Cardiol. (2025) 85:106–16. doi: 10.1016/j.jacc.2024.09.004 39352340

[38] Anker SD Butler J Filippatos G Ferreira JP Bocchi E Böhm M . Empagliflozin in heart failure with a preserved ejection fraction. N Engl J Med. (2021) 385:1451–61. doi: 10.1056/NEJMoa2107038 34449189

[39] Solomon SD McMurray JJV Claggett B de Boer RA DeMets D Hernandez AF . Dapagliflozin in heart failure with mildly reduced or preserved ejection fraction. N Engl J Med. (2022) 387:1089–98. doi: 10.1056/NEJMoa2206286 36027570

[40] Verma S McMurray JJV . SGLT2 inhibitors and mechanisms of cardiovascular benefit: a state-of-the-art review. Diabetologia. (2018) 61:2108–17. doi: 10.1007/s00125-018-4670-7 30132036

[41] McDowell K Docherty KF Campbell RT Henderson AD Jhund PS Claggett BL . Finerenone for heart failure and risk estimated by the PREDICT-HFpEF model: a secondary analysis of FINEARTS-HF. JAMA Cardiol. (2025) 10:535–44. doi: 10.1001/jamacardio.2025.0025 40042880 PMC11883611

[42] Chen Y Jiang M Xia C Zhao H Ke P Chen S . A novel deep learning system for STEMI prognostic prediction from multi-sequence cardiac magnetic resonance. Sci Bull (Beijing). (2025) 70:4241–52. doi: 10.1016/j.scib.2025.11.027 41314962

[43] Wolff RF Moons KGM Riley RD Whiting PF Westwood M Collins GS . PROBAST: a tool to assess the risk of bias and applicability of prediction model studies. Ann Intern Med. (2019) 170:51–8. doi: 10.7326/M18-1376 30596875

[44] Van Calster B McLernon DJ van Smeden M Wynants L Steyerberg EW . Calibration: the Achilles heel of predictive analytics. BMC Med. (2019) 17:230. doi: 10.1186/s12916-019-1466-7 31842878 PMC6912996

[45] Obokata M Reddy YNV Pislaru SV Melenovsky V Borlaug BA . Evidence supporting the existence of a distinct obese phenotype of heart failure with preserved ejection fraction. Circulation. (2017) 136:6–19. doi: 10.1161/CIRCULATIONAHA.116.026807 28381470 PMC5501170

