# Supplementary material for: A clinical prediction model integrating cardiovascular-kidney-metabolic biomarkers for the composite outcome of quality-of-life deterioration and rehospitalization in elderly HFpEF patients
Source: Front Endocrinol (Lausanne). 2026 Aug 3;17:1861971. doi: 10.3389/fendo.2026.1861971 (PMC13477927; doi:10.3389/fendo.2026.1861971)
Supplement: Supplementary file 1 [file Image1.pdf]

figure s1

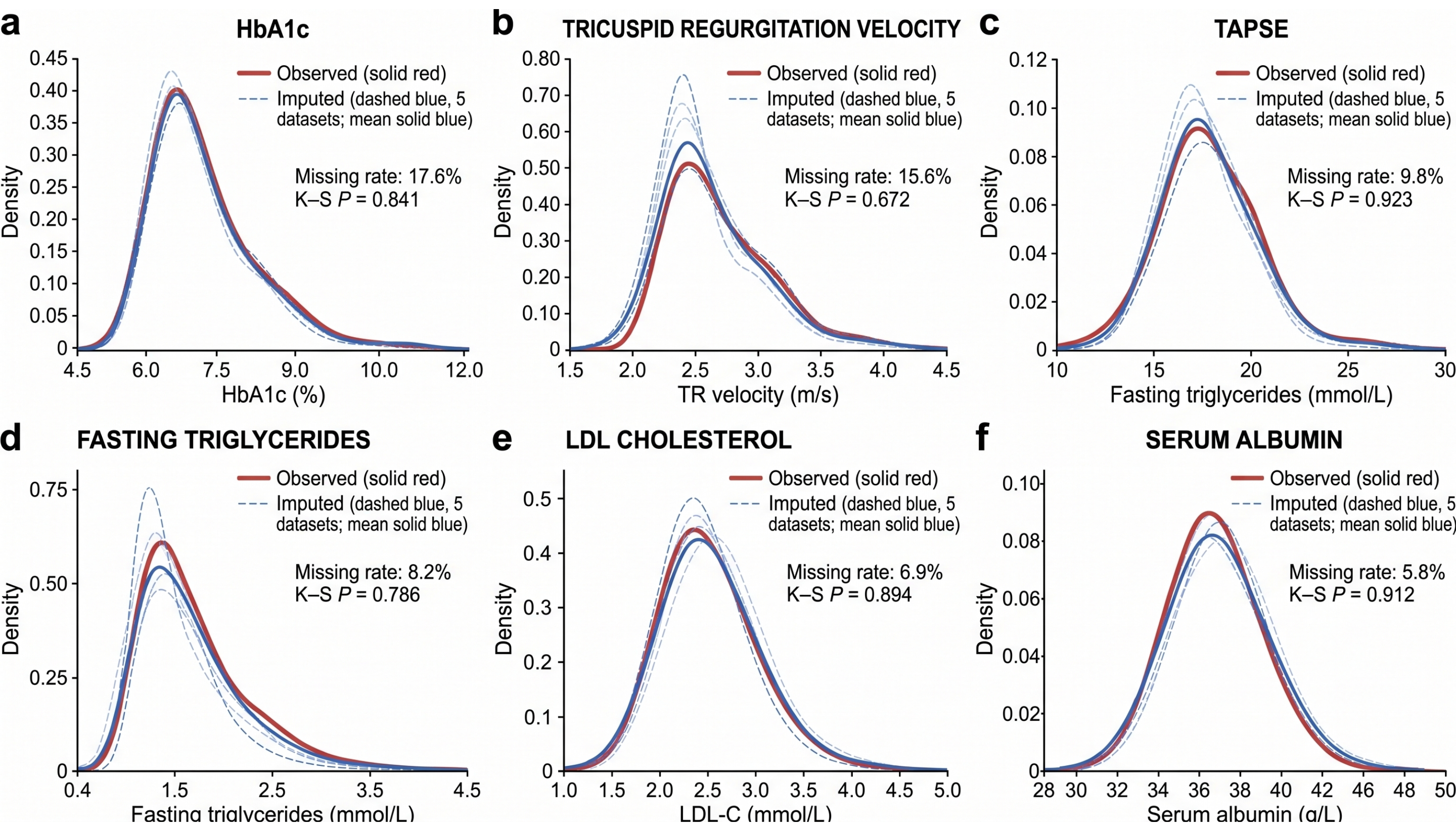

a
HbA1c
Observed (solid red)
Imputed (dashed blue, 5 datasets; mean solid blue)
Missing rate: 17.6%
K–S P = 0.841
Density
HbA1c (%)
b
TRICUSPID REGURGITATION VELOCITY
Observed (solid red)
Imputed (dashed blue, 5 datasets; mean solid blue)
Missing rate: 15.6%
K–S P = 0.672
Density
TR velocity (m/s)
c
TAPSE
Observed (solid red)
Imputed (dashed blue, 5 datasets; mean solid blue)
Missing rate: 9.8%
K–S P = 0.923
Density
Fasting triglycerides (mmol/L)
d
FASTING TRIGLYCERIDES
Observed (solid red)
Imputed (dashed blue, 5 datasets; mean solid blue)
Missing rate: 8.2%
K–S P = 0.786
Density
Fasting triglycerides (mmol/L)
e
LDL CHOLESTEROL
Observed (solid red)
Imputed (dashed blue, 5 datasets; mean solid blue)
Missing rate: 6.9%
K–S P = 0.894
Density
LDL-C (mmol/L)
f
SERUM ALBUMIN
Observed (solid red)
Imputed (dashed blue, 5 datasets; mean solid blue)
Missing rate: 5.8%
K–S P = 0.912
Density
Serum albumin (g/L)

figure s2

LASSO-Cox variable selection stability across five multiply imputed datasets (λ.min model, n = 302)

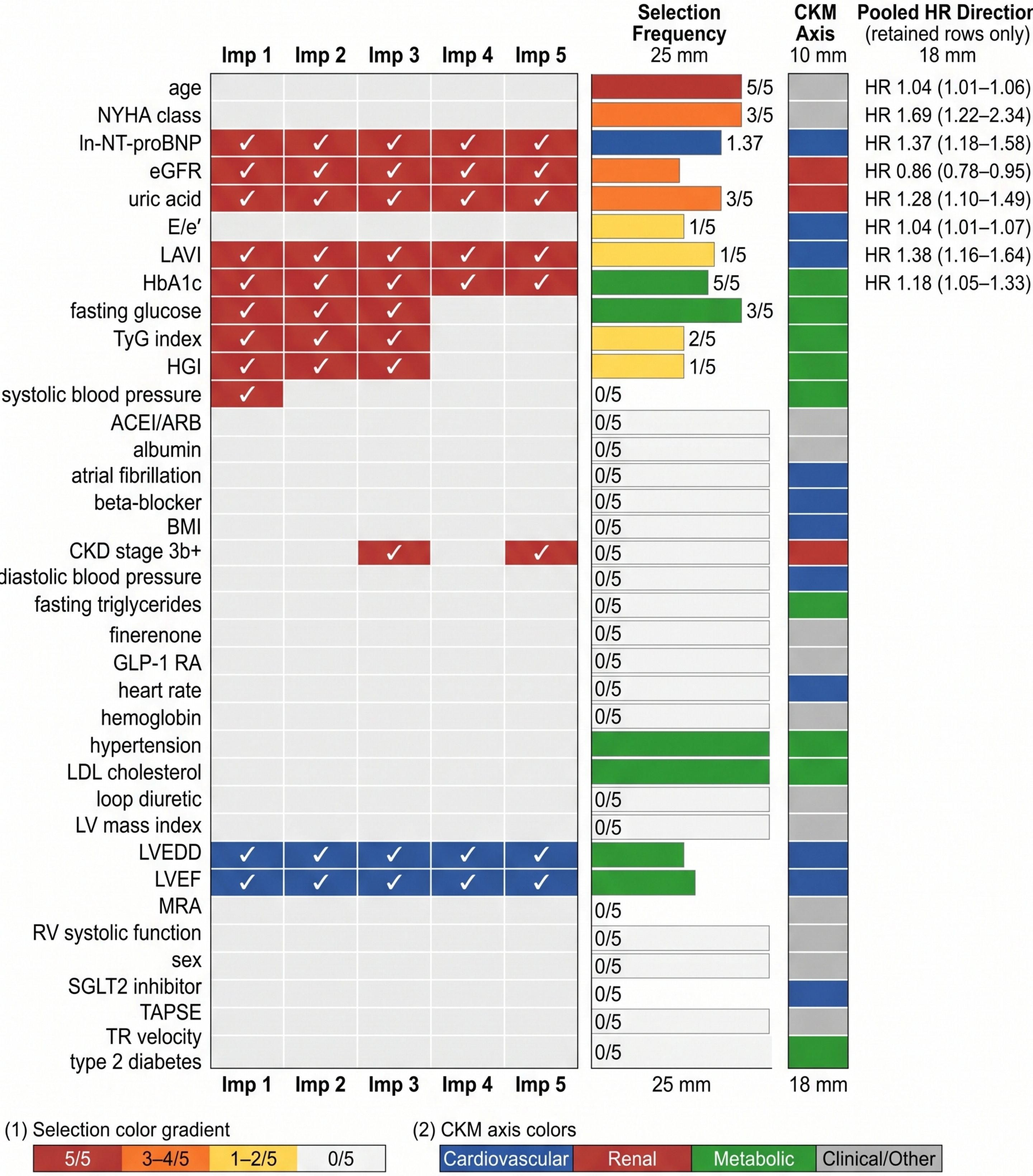

(3) Pooled HR shown for retained predictors only; HR estimates pooled across 5 imputations by Rubin's rules.

figure s3

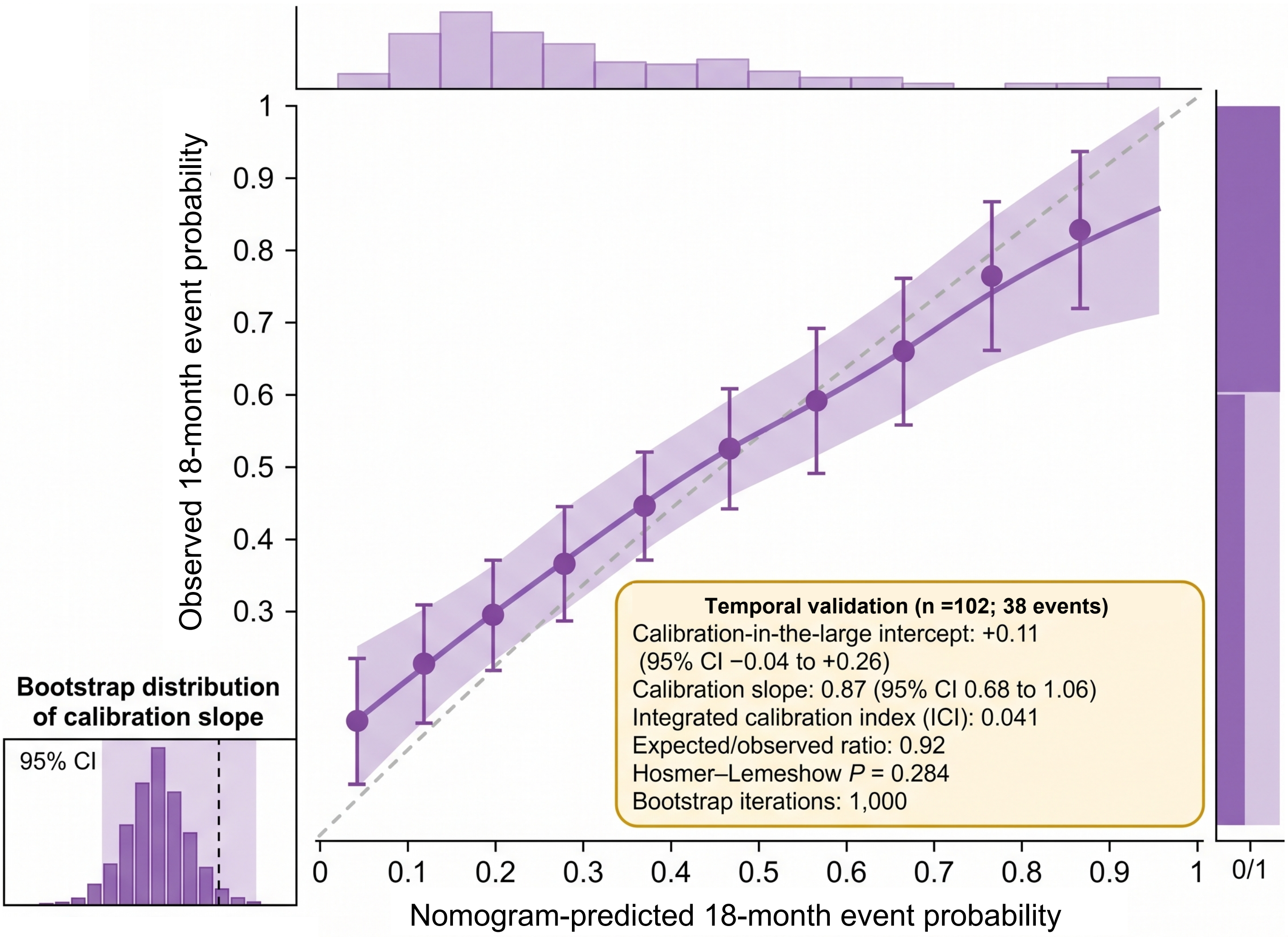
Bootstrap distribution of calibration slope
95% CI
Observed 18-month event probability
1
0.9
0.8
0.7
0.6
0.5
0.4
0.3
0
0.1
0.2
0.3
0.4
0.5
0.6
0.7
0.8
0.9
1
0/1
Nomogram-predicted 18-month event probability
Temporal validation (n =102; 38 events)
Calibration-in-the-large intercept: +0.11
(95% CI −0.04 to +0.26)
Calibration slope: 0.87 (95% CI 0.68 to 1.06)
Integrated calibration index (ICI): 0.041
Expected/observed ratio: 0.92
Hosmer–Lemeshow P = 0.284
Bootstrap iterations: 1,000
