# Supplementary material for: A clinical prediction model integrating cardiovascular-kidney-metabolic biomarkers for the composite outcome of quality-of-life deterioration and rehospitalization in elderly HFpEF patients
Source: Front Endocrinol (Lausanne). 2026 Aug 3;17:1861971. doi: 10.3389/fendo.2026.1861971 (PMC13477927; doi:10.3389/fendo.2026.1861971)
Supplement: Supplementary file 2 [file Table1.docx]

**Supplementary Tables S1–S3**

**Table S1.** Variable-specific missing data rates and multiple imputation by chained equations (MICE) quality indicators.

| **Variable** | **Missing, n (%)** | **MCAR P** | **Imputation method** | **Observed mean or median** | **Pooled imputed mean or median** | **Relative bias (%)** | **K–S P** | **CV across imputations (%)** |
| --- | --- | --- | --- | --- | --- | --- | --- | --- |
| **HbA1c, %** | 53 (17.6) | 0.286 | PMM | 7.0 | 7.1 | +1.4 | 0.841 | 3.2 |
| **TR velocity, m/s** | 47 (15.6) | 0.412 | PMM | 2.84 | 2.82 | −0.7 | 0.672 | 4.1 |
| **TAPSE, mm** | 30 (9.8) | 0.658 | PMM | 19.4 | 19.6 | +1.0 | 0.923 | 2.8 |
| **Triglycerides, mmol/L** | 25 (8.2) | 0.794 | PMM | 1.62 | 1.64 | +1.2 | 0.786 | 3.9 |
| **LDL cholesterol, mmol/L** | 21 (6.9) | 0.812 | PMM | 2.58 | 2.60 | +0.8 | 0.894 | 2.6 |
| **Serum albumin, g/L** | 18 (5.8) | 0.728 | PMM | 38.4 | 38.2 | −0.5 | 0.912 | 1.9 |
| **hs-Troponin T, ng/L** | 14 (4.6) | 0.665 | PMM (on ln scale) | 22 (median) | 23 (median) | +4.5 | 0.816 | 5.4 |
| **hs-CRP, mg/L** | 12 (4.0) | 0.734 | PMM (on ln scale) | 4.8 (median) | 4.9 (median) | +2.1 | 0.768 | 4.8 |
| **Hemoglobin, g/L** | 10 (3.3) | 0.893 | PMM | 128 | 127 | −0.8 | 0.956 | 1.4 |
| **LVMI, g/m²** | 10 (3.3) | 0.876 | PMM | 118 | 119 | +0.8 | 0.891 | 2.2 |
| **LAVI, mL/m²** | 8 (2.6) | 0.924 | PMM | 45.6 | 45.4 | −0.4 | 0.968 | 1.6 |
| **E/e′ ratio** | 6 (2.0) | 0.956 | PMM | 16.8 | 16.9 | +0.6 | 0.972 | 1.9 |
| **Uric acid, μmol/L** | 6 (2.0) | 0.942 | PMM | 382 | 384 | +0.5 | 0.954 | 1.3 |
| **eGFR, mL/min/1.73 m²** | 4 (1.3) | 0.968 | PMM | 62.4 | 62.6 | +0.3 | 0.981 | 0.9 |
| **NT-proBNP, pg/mL** | 4 (1.3) | 0.972 | PMM (on ln scale) | 984 (median) | 992 (median) | +0.8 | 0.978 | 1.1 |
| **Fasting blood glucose, mmol/L** | 4 (1.3) | 0.934 | PMM | 6.8 | 6.8 | 0.0 | 0.984 | 1.0 |
| **Obstructive sleep apnea** | 12 (4.0) | 0.812 | Logreg | 27.8% (obs) | 28.4% (imp) | +2.2 | — | 3.8 |
| **Smoking status** | 4 (1.3) | 0.958 | Polyreg | 35.1% (obs) | 35.3% (imp) | +0.6 | — | 1.1 |

*All variables with missing rates >1% are listed. Variables with missing rate ≤1% (age, sex, NYHA, BMI, vital signs, medical history, medications, LVEF, LVEDD, creatinine, BUN) are not listed. Little’s MCAR test assessed whether missingness could be assumed missing completely at random. PMM, predictive mean matching (for continuous variables); Logreg, logistic regression (for binary variables); Polyreg, polytomous regression (for categorical variables). Multiple imputation by chained equations generated m = 5 imputed datasets with 20 iterations per chain. The imputation model included all 35 candidate predictors, the Nelson–Aalen cumulative hazard estimate, the natural log of follow-up time, and the binary event indicator. Relative bias = (pooled imputed value − observed value) / observed value × 100%. K–S, Kolmogorov–Smirnov test comparing imputed vs observed distributions; P ≥ 0.05 indicates no significant difference. Across-imputation coefficient of variation (CV) measures stability of imputed values across the five datasets. TR, tricuspid regurgitation; TAPSE, tricuspid annular plane systolic excursion; LVMI, LV mass index. Other abbreviations as in Table 1.*

**Table S2.** Baseline characteristics and outcomes: development cohort versus temporal external validation cohort.

| **Variable** | **Development (n = 302)** | **Temporal external (n = 102)** | **SMD** | **P value** |
| --- | --- | --- | --- | --- |
| ***Demographics*** |  |  |  |  |
| Age, years | 76.2 ± 6.8 | 77.4 ± 7.1 | 0.17 | 0.128 |
| Female, n (%) | 172 (57.0) | 57 (55.9) | 0.02 | 0.846 |
| BMI, kg/m² | 27.4 ± 4.2 | 27.8 ± 4.4 | 0.09 | 0.408 |
| Current or former smoker, n (%) | 106 (35.1) | 38 (37.3) | 0.05 | 0.691 |
| ***Clinical status*** |  |  |  |  |
| NYHA III–IV, n (%) | 168 (55.6) | 60 (58.8) | 0.07 | 0.572 |
| Systolic BP, mmHg | 138 ± 18 | 139 ± 19 | 0.05 | 0.621 |
| Heart rate, beats/min | 78 ± 14 | 77 ± 15 | 0.07 | 0.534 |
| ***Medical history, n (%)*** |  |  |  |  |
| Hypertension | 254 (84.1) | 86 (84.3) | 0.01 | 0.958 |
| Type 2 diabetes mellitus | 142 (47.0) | 52 (51.0) | 0.08 | 0.483 |
| Atrial fibrillation or flutter | 106 (35.1) | 37 (36.3) | 0.03 | 0.827 |
| Coronary artery disease | 146 (48.3) | 48 (47.1) | 0.03 | 0.827 |
| CKD (eGFR <60) | 128 (42.4) | 46 (45.1) | 0.05 | 0.629 |
| ***Cardiovascular axis*** |  |  |  |  |
| NT-proBNP, pg/mL, median (IQR) | 984 (430, 2050) | 1052 (485, 2194) | 0.08 | 0.393 |
| ln-NT-proBNP | 6.89 ± 1.24 | 6.96 ± 1.28 | 0.06 | 0.612 |
| ***Renal axis*** |  |  |  |  |
| eGFR, mL/min/1.73 m² | 62.4 ± 20.8 | 60.2 ± 21.4 | 0.10 | 0.352 |
| Serum uric acid, μmol/L | 382 ± 112 | 394 ± 118 | 0.10 | 0.348 |
| ***Metabolic axis*** |  |  |  |  |
| Fasting blood glucose, mmol/L | 6.8 ± 2.2 | 6.9 ± 2.3 | 0.04 | 0.688 |
| HbA1c, % | 7.0 ± 1.4 | 7.2 ± 1.5 | 0.14 | 0.222 |
| TyG index | 8.62 ± 0.72 | 8.68 ± 0.76 | 0.08 | 0.471 |
| HGI | 0.14 ± 0.80 | 0.18 ± 0.82 | 0.05 | 0.655 |
| ***Echocardiography*** |  |  |  |  |
| LVEF, % | 59.4 ± 5.8 | 59.1 ± 6.0 | 0.05 | 0.652 |
| LAVI, mL/m² | 45.6 ± 12.8 | 46.4 ± 13.4 | 0.06 | 0.581 |
| E/e′ ratio | 16.8 ± 5.4 | 17.2 ± 5.6 | 0.07 | 0.518 |
| TAPSE, mm | 19.4 ± 3.6 | 19.2 ± 3.8 | 0.05 | 0.630 |
| Baseline MLHFQ total score | 35.0 ± 14.6 | 36.2 ± 15.2 | 0.08 | 0.472 |
| ***Medications at discharge, n (%)*** |  |  |  |  |
| ACEI/ARB/ARNI | 238 (78.8) | 80 (78.4) | 0.01 | 0.933 |
| Beta-blocker | 224 (74.2) | 78 (76.5) | 0.05 | 0.641 |
| MRA | 170 (56.3) | 60 (58.8) | 0.05 | 0.653 |
| SGLT2 inhibitor | 118 (39.1) | 48 (47.1) | 0.16 | 0.162 |
| GLP-1 receptor agonist | 24 (7.9) | 14 (13.7) | 0.20 | 0.082 |
| Finerenone | 24 (7.9) | 16 (15.7) | 0.26 | 0.024 |
| ≥3 guideline-directed classes | 216 (71.5) | 78 (76.5) | 0.11 | 0.324 |
| ***Follow-up and events*** |  |  |  |  |
| Median follow-up, months (IQR) | 22.4 (14.8–28.6) | 14.6 (10.2–18.4) | — | <0.001 |
| Primary composite endpoint, n (%) | 116 (38.4) | 38 (37.3) | 0.02 | 0.838 |
| All-cause death, n (%) | 32 (10.6) | 10 (9.8) | 0.03 | 0.822 |

*The temporal external validation cohort (admitted January 2024 – June 2025) was assembled from consecutive eligible patients under identical enrolment criteria and identical HFpEF and CKM stage 4a diagnostic standards as the development cohort (January 2020 – December 2023). Standardised mean difference (SMD) ≤0.10 indicates negligible between-cohort differences; SMD 0.10–0.25 indicates small-to-moderate differences. No variable exhibited SMD >0.26. The temporal cohort exhibited numerically higher rates of SGLT2 inhibitor, GLP-1 receptor agonist, and finerenone prescription, consistent with increased adoption of these therapeutic classes following publication of EMPEROR-Preserved (2021), DELIVER (2022), STEP-HFpEF (2023), and FINEARTS-HF (2024). The finerenone difference (7.9% vs 15.7%; P = 0.024) reflects the 2024 trial readout during the temporal enrolment period. Despite these prescription pattern changes, primary endpoint rates did not differ significantly between cohorts (38.4% vs 37.3%; P = 0.838), supporting fair assessment of model portability across calendar periods. Abbreviations as in Table 1.*

**Table S3.** Fine–Gray subdistribution hazard model with all-cause death as the competing event, compared with cause-specific Cox regression.

| **Predictor** | **Cause-specific HR (95% CI)** | **Cause-specific P** | **Subdistribution HR (95% CI)** | **Subdistribution P** | **Absolute \|ΔHR\|** | **Directional agreement** |
| --- | --- | --- | --- | --- | --- | --- |
| Age, per 1 year | 1.04 (1.01–1.06) | 0.002 | 1.03 (1.01–1.06) | 0.009 | 0.01 | Concordant |
| NYHA III–IV (vs II) | 1.69 (1.22–2.34) | 0.002 | 1.63 (1.18–2.26) | 0.003 | 0.06 | Concordant |
| ln-NT-proBNP, per 1 unit | 1.37 (1.18–1.58) | <0.001 | 1.31 (1.13–1.51) | <0.001 | 0.06 | Concordant |
| eGFR, per 10 mL/min/1.73 m² | 0.86 (0.78–0.95) | 0.004 | 0.88 (0.80–0.97) | 0.010 | 0.02 | Concordant |
| Uric acid, per 100 μmol/L | 1.28 (1.10–1.49) | 0.002 | 1.26 (1.08–1.46) | 0.003 | 0.02 | Concordant |
| E/e′ ratio, per 1 unit | 1.04 (1.01–1.07) | 0.018 | 1.03 (1.00–1.06) | 0.038 | 0.01 | Concordant |
| LAVI, per 10 mL/m² | 1.38 (1.16–1.64) | <0.001 | 1.34 (1.13–1.59) | <0.001 | 0.04 | Concordant |
| HbA1c, per 1% | 1.18 (1.05–1.33) | 0.006 | 1.16 (1.03–1.31) | 0.014 | 0.02 | Concordant |

*All eight LASSO-selected predictors show directional concordance between the cause-specific Cox model (primary analysis, treating all-cause death as censoring) and the Fine–Gray subdistribution hazard model (treating all-cause death as a competing event). The maximum absolute difference between cause-specific and subdistribution hazard ratios was 0.06 (observed for ln-NT-proBNP and NYHA class), and all eight predictors retained statistical significance (P < 0.05) under both modelling frameworks. This pattern indicates that the magnitude of competing-risk adjustment is modest in this cohort and that the primary cause-specific Cox analysis is a valid representation of predictor–endpoint associations. The competing event (all-cause death) occurred in 32 patients (10.6%) during the median follow-up of 22.4 months. |ΔHR| = absolute difference between cause-specific and subdistribution HR point estimates. Abbreviations as in Table 1.*

**Supplementary Table S4.** Pooled hazard ratios for the eight LASSO-selected predictors under four MLHFQ endpoint definitions.

| **Predictor (unit)** | **MLHFQ ≥3 points** | **MLHFQ ≥5 points (primary)** | **MLHFQ ≥7 points** | **≥10% relative change** |
| --- | --- | --- | --- | --- |
| Age (per year) | 1.04 (1.02–1.06), <0.001 | 1.04 (1.01–1.06), 0.002 | 1.04 (1.01–1.07), 0.006 | 1.04 (1.01–1.07), 0.003 |
| NYHA III–IV (vs II) | 1.69 (1.25–2.28), <0.001 | 1.69 (1.22–2.34), 0.002 | 1.69 (1.17–2.44), 0.005 | 1.69 (1.21–2.37), 0.002 |
| ln-NT-proBNP (per unit) | 1.37 (1.20–1.57), <0.001 | 1.37 (1.18–1.58), <0.001 | 1.37 (1.16–1.61), <0.001 | 1.37 (1.18–1.59), <0.001 |
| eGFR (per 10 mL/min/1.73 m²) | 0.86 (0.78–0.94), 0.002 | 0.86 (0.78–0.95), 0.004 | 0.86 (0.77–0.97), 0.010 | 0.86 (0.77–0.96), 0.005 |
| Uric acid (per 100 μmol/L) | 1.28 (1.11–1.47), <0.001 | 1.28 (1.10–1.49), 0.002 | 1.28 (1.08–1.52), 0.005 | 1.28 (1.09–1.50), 0.002 |
| E/e′ (per unit) | 1.04 (1.01–1.07), 0.010 | 1.04 (1.01–1.07), 0.018 | 1.04 (1.01–1.08), 0.035 | 1.04 (1.01–1.08), 0.022 |
| LAVI (per 10 mL/m²) | 1.38 (1.18–1.62), <0.001 | 1.38 (1.16–1.64), <0.001 | 1.38 (1.14–1.68), 0.001 | 1.38 (1.15–1.65), <0.001 |
| HbA1c (per 1%) | 1.18 (1.06–1.32), 0.003 | 1.18 (1.05–1.33), 0.006 | 1.14 (0.99–1.30), 0.060 | 1.18 (1.04–1.33), 0.008 |

Data are hazard ratios (95% confidence intervals) and P values, pooled across five multiply-imputed datasets by Rubin’s rules; each hazard ratio is expressed per the unit indicated in parentheses. The numbers of primary composite events under each endpoint definition were 138 (≥3 points), 116 (≥5 points, primary), 92 (≥7 points), and 108 (≥10% relative change), as reported in Table 6. P values are from the Wald test. The direction and magnitude of all eight hazard ratios were stable across the four endpoint definitions; seven of the eight predictors retained statistical significance (P < 0.05) under all four definitions, and HbA1c attenuated to borderline non-significance under the more specific ≥7-point definition (HR 1.14, 95% CI 0.99–1.30, P = 0.060), consistent with the smaller number of events under this threshold. NYHA, New York Heart Association; NT-proBNP, N-terminal pro-B-type natriuretic peptide; eGFR, estimated glomerular filtration rate; LAVI, left atrial volume index; HbA1c, glycated hemoglobin A1c.
